# Nutrient Element Decorated Polyetheretherketone Implants Steer Mitochondrial Dynamics for Boosted Diabetic Osseointegration

**DOI:** 10.1002/advs.202101778

**Published:** 2021-08-16

**Authors:** Hao Wang, Xinliang Fu, Jiacheng Shi, Limei Li, Jiyu Sun, Xidan Zhang, Qiuyang Han, Yi Deng, Xueqi Gan

**Affiliations:** ^1^ State Key Laboratory of Oral Diseases National Clinical Research Center for Oral Diseases West China Hospital of Stomatology Sichuan University Chengdu 610041 China; ^2^ School of Chemical Engineering State Key Laboratory of Polymer Materials Engineering Sichuan University Chengdu 610065 China; ^3^ Science and Technology Achievement Incubation Center Kunming Medical University Kunming 650500 China; ^4^ Department of Mechanical Engineering The University of Hong Kong Hong Kong SAR China

**Keywords:** mitochondrial dynamics, nutrient element, orthopedic implant, osseointegration, polyetheretherketone

## Abstract

As a chronic metabolic disease, diabetes mellitus (DM) creates a hyperglycemic micromilieu around implants, resulting inthe high complication and failure rate of implantation because of mitochondrial dysfunction in hyperglycemia. To address the daunting issue, the authors innovatively devised and developed mitochondria‐targeted orthopedic implants consisted of nutrient element coatings and polyetheretherketone (PEEK). Dual nutrient elements, in the modality of ZnO and Sr(OH)_2_, are assembled onto the sulfonated PEEK surface (Zn&Sr‐SPEEK). The results indicate the synergistic liberation of Zn^2+^ and Sr^2+^ from coating massacres pathogenic bacteria and dramatically facilitates cyto‐activity of osteoblasts upon the hyperglycemic niche. Intriguingly, Zn&Sr‐SPEEK implants are demonstrated to have a robust ability to recuperate hyperglycemia‐induced mitochondrial dynamic disequilibrium and dysfunction by means of Dynamin‐related protein 1 (Drp1) gene down‐regulation, mitochondrial membrane potential (MMP) resurgence, and reactive oxygen species (ROS) elimination, ultimately enhancing osteogenicity of osteoblasts. In vivo evaluations utilizing diabetic rat femoral/tibia defect model at 4 and 8 weeks further confirm that nutrient element coatings substantially augment bone remodeling and osseointegration. Altogether, this study not only reveals the importance of Zn^2+^ and Sr^2+^ modulation on mitochondrial dynamics that contributes to bone formation and osseointegration, but also provides a novel orthopedic implant for diabetic patients with mitochondrial modulation capability.

## Graphical abstract

The synthesized dual nutrient element decorated implants enable to inhibit the overexpression of Drp1 gene, eliminate accumulated ROS of cells, recuperate MMP, ultimately contributing to regulating mitochondrial dynamics and functions, thereby enhancing osteogenicity of osteoblasts in hyperglycemia, which hold considerable promise for the future design of mitochondria‐targeted pathological implants.

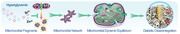

## 1 Introduction

Diabetes mellitus, characterized by hyperglycemia due to insulin insensitivity or deficiency,^[^ ^1^ ^]^ is suffered by 463 million people in the age range of 20 to 79 years in 2019, while the number is projected to increase to ≈578 million by 2030.^[^ ^2^ ^]^ Type 1 DM (T1DM)‐associated skeletal complications,^[^ ^3^ ^]^ including osteopenia and inferior fracture/defect healing, for which effective methods are still lacking, have been becoming an emerging clinical challenge for the whole world. Evidence from both the bench and the bedside has confirmed that chronic hyperglycemia impairs osseointegration of implants resulting from osteoblast dysfunction behavior and bone turnover inhibition.^[^ ^4^ ^]^ All of the above directly influence clinical efficacy and lead to a higher rate of implant loosening or failure. Although current implant materials through surface modification have committed to optimizing osseointegration,^[^ ^5^ ^]^ including calcium phosphates, biomolecules, hydroxyapatite, and tantalum decorated, their efficacy in DM is disappointing with a high complication and failure rate partly due to mitochondrial dysfunction in hyperglycemic micromilieu around implants. Therefore, exploring a more satisfying solution for osseointegration under DM is still a long way to walk.

Mitochondria are subcellular organelles in the form of a dynamic, interconnected network.^[^ ^6^ ^]^ Characterized by constant fission, fusion, and trafficking, mitochondrial dynamic equilibrium controls both the ultrastructure and functions of mitochondria^[^ ^7^ ^]^ and serves as an essential driver for sustaining metabolic fitness in response to metabolic perturbations.^[^ ^8^ ^]^ Mitochondrial dynamics is involved in the maintenance and commitment of stem cells^[^ ^9^ ^]^ and performs a crucial function in the osteogenic differentiation of osteoblasts.^[^ ^10^ ^]^ Recent discoveries remind us that osteogenicity can be disrupted by mitochondrial dynamic disorders induced by oxidative stress, physical vibration, and genetic mutation.^[^ ^11^ ^]^ Mitochondrial dynamic equilibrium, impaired by a sustained hyperglycemic micromilieu,^[^ ^12^ ^]^ has been indicated as upstream events leading to DM and its complications in bone remodeling.^[^ ^13^ ^]^ Additionally, mitochondria serve as the primary source of ROS in cells, and ROS are conversely potent stimulants of mitochondrial fission.^[^ ^14^ ^]^ Excessive ROS has been demonstrated to induce mitochondrial fragmentation,^[^ ^10^, ^15^ ^]^ amplifying the initial oxidative stress via the process termed as “ROS‐induced ROS release.”^[^ ^16^ ^]^ However, the excessive ROS generation mediated by diabetes is implicated in bone regeneration and compromises implant osseointegration.^[^ ^17^ ^]^ Previous studies by our group have indicated that Drp1, which regulates mitochondrial fission, is increased in high doses of d‐glucose and results in ROS overgeneration and a change of mitochondrial morphologies from a continuous network to fragmentation, contributing to an imbalance in mitochondrial dynamics.^[^ ^18^ ^]^ Recently, explorations of treatment strategies targeting mitochondrial function have drawn wide concern.^[^ ^19^ ^]^ Mitochondria‐targeted antioxidant^[^ ^20^ ^]^ can notably inhibit bone loss in diabetic and high fat diet‐fed mice. Besides, pharmacological and genetic inhibition of mitophagy^[^ ^21^ ^]^ can reverse dysfunction in osteoblastic cells.^[^ ^22^ ^]^ Therefore, a mitochondria‐targeted biomaterial that can regulate mitochondrial dynamic and functional alterations result from hyperglycemia and construct an optimal intracellular microenvironment for natural ossification should holds considerable promise in exploring a new strategy for osteogenesis of an implant.

The bone of our body contains various mineral compositions and trace nutritional elements, such as Mg^2+^, Zn^2+^, and Sr^2+^, which keep balance in the local microenvironment during bone metabolism.^[^ ^23^ ^]^ Sr plays a dual‐acting role in bone reconstruction, inhibiting bone resorption and stimulating bone formation.^[^ ^24^ ^]^ The majority of animal experiments and clinical trials support that Sr significantly augments bone density and reduces osteoporotic bone loss, benefiting the therapeutic effect of osteoporosis.^[^ ^25^ ^]^ Furthermore, Sr is also associated with other biological processes such as angiogenesis^[^ ^26^ ^]^ and osteoimmunomodulation,^[^ ^27^ ^]^ which work together in promoting osteogenesis. Accounting for a large proportion of body skeleton, Zn has been acknowledged to have an essential role in the formation and mineralization of bone.^[^ ^28^ ^]^ It is identified that zinc participates in controlling alkaline phosphatase (ALP) activity and mineralization process.^[^ ^29^ ^]^ Furthermore, a proper supply of Zn^2+^ can protect mitochondria from oxidative damage,^[^ ^30^ ^]^ which is important for homeostasis, differentiation, and maturation of osteoblast.^[^ ^31^ ^]^ Although previous studies have reported that integrating Sr or Zn into orthopedic materials enable to promote osseointegration in vivo,^[^ ^32^ ^]^ there are few reports concerning mitochondria‐targeted biomaterial‐mediated bone regeneration in DM.

Benefiting from excellent mechanical properties, chemical resistance, and natural radiolucency, polyetheretherketone (PEEK) has become the leading materials in orthopedics, dentistry, and traumatology.^[^ ^33^ ^]^ Also, Young's modulus of PEEK is closer to that of cortical bone, which reduces the stress shielding effect in the progress of implant and alleviates the rate of premature implant failure and around bone resorption.^[^ ^34^ ^]^ Earlier literature has reported that metal ion or hydroxyapatite modification enables to render polymer scaffolds with antibacterial activity and osteogenecity.^[^ ^35^ ^]^ Taking the foregoing consideration, herein, we designed and developed a multifunctional PEEK orthopedic implant decorated with dual nutrient elements Zn and Sr through metal ion modification for steering mitochondrial dynamics and functions. Our study aims at rescuing the mitochondrial dysfunction and accelerating osteogenic differentiation of osteoblasts in hyperglycemia, as well as facilitating diabetic bone remolding in vivo. We struggle to elucidate the relationship between mitochondrial dynamics and the osseointegration of implants. It is expected to provide an avenue for diabetic bone repairing through mitochondria‐targeted biomaterial‐enabled bone regeneration therapy.

## 2 Results and Discussion

### 2.1 Preparation and Characterization of Nutrient Element Coatings on PEEK Implant

**Scheme 1:**
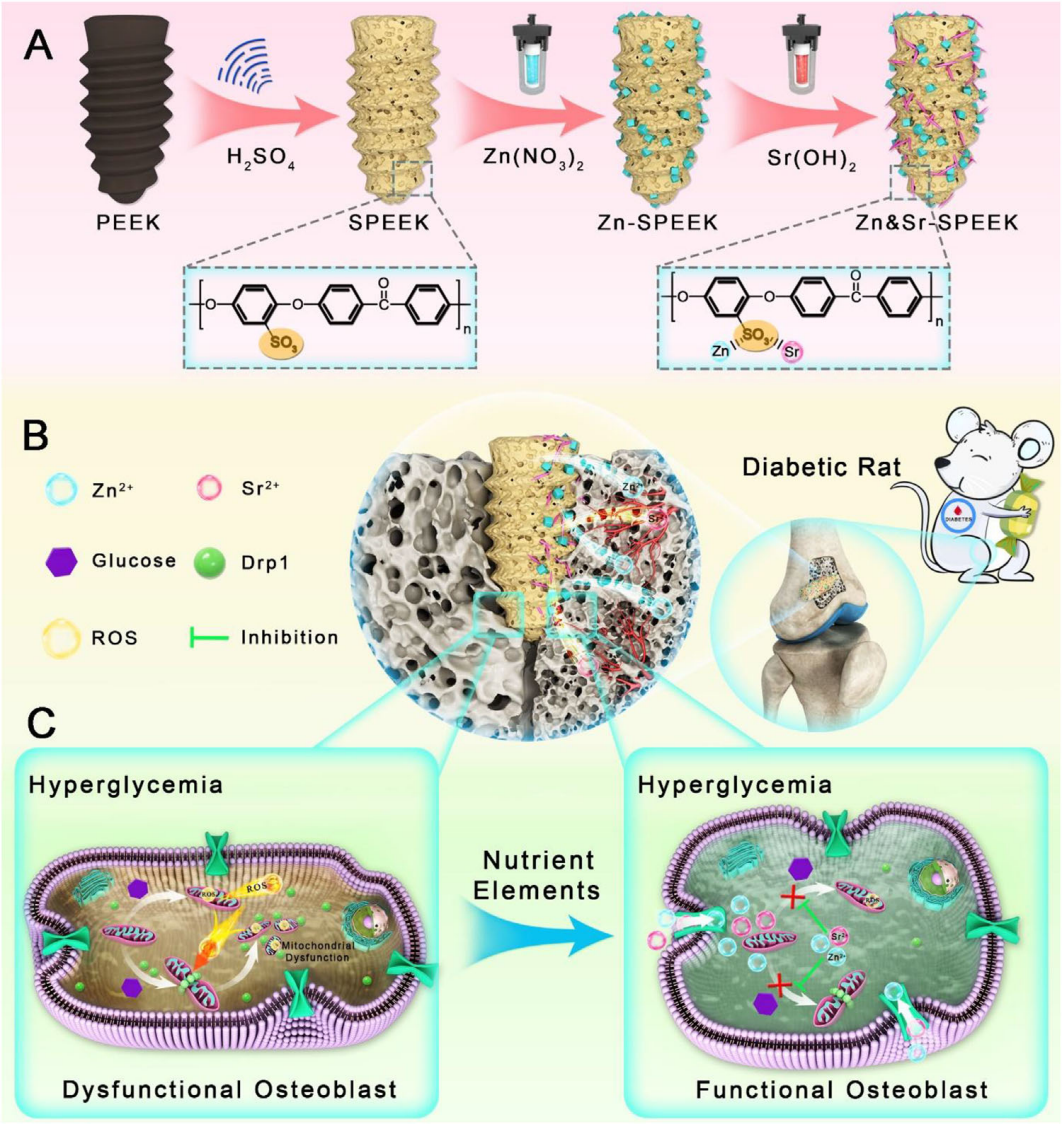
Schematic illustration for A) the preparation of Zn and Sr nutrient element‐decorated PEEK implants, B) diabetic osseointegration process of the functionalized PEEK implants in vivo, and C) the mitochondrial‐targeted osteogenic pathway of the Zn&Sr‐SPEEK implants.

To tackle the high complication and failure rate of implantation in DM, we innovatively devise a mitochondria‐targeted orthopedic implant consisting of nutrient element coatings and PEEK imparted with potent regulation of mitochondrial dynamics and superior osteogenicity upon hyperglycemic micromilieu. As shown in **Scheme** **1**A, ZnO and Sr(OH)_2_ nanoparticles are grown firmly onto the SPEEK through the electrostatic interaction between negatively polarized groups (—SO_3_) of SPEEK with positively charged Zn(II) and Sr(II) during the hydrothermal process, ultimately constructing dual nutrient elements decorated orthopedic implants. Compared with the reported chemical conversion coating (Sr‐Zn phosphate coating) which is commonly fabricated on metallic surfaces, the potential advantage of the dual nutrient elements (Zn&Sr) coating is its versatility in modification of various implants made from metals, ceramics and polymers.^[^ ^36^ ^]^

**Figure 1:**
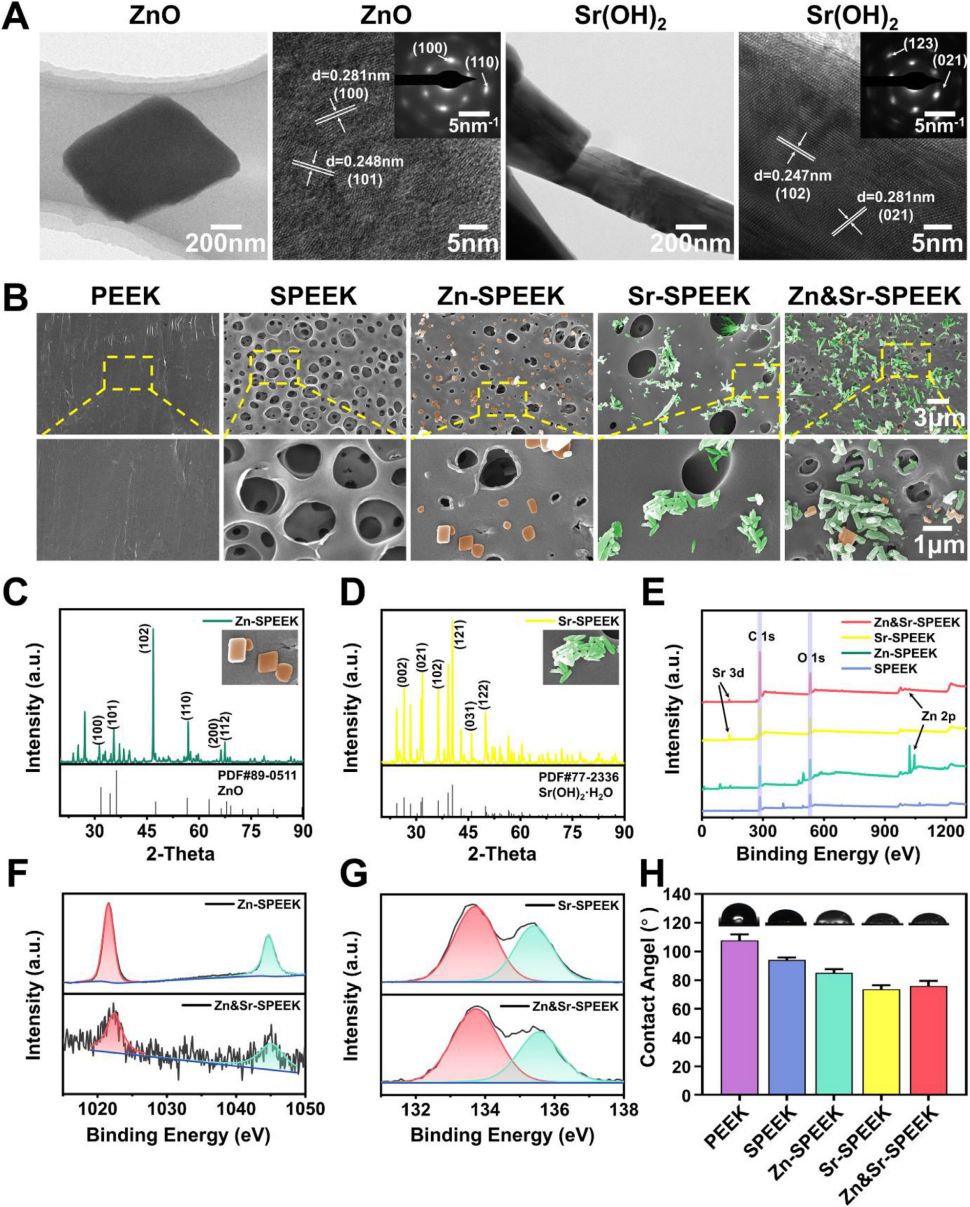
Characterization of ZnO, Sr(OH)_2_, and functionalized PEEK: A) TEM, HR‐TEM images and SAED patterns of ZnO and Sr(OH)_2_ nanoparticles; B) SEM microscopy images of PEEK and different decorated SPEEK. The materials compositions are colored for visual observation (orange, ZnO crystals; green, Sr(OH)_2_ crystals); XRD patterns of C) Zn‐SPEEK and D)Sr‐SPEEK; E) XPS analysis of SPEEK, Zn‐SPEEK, Sr‐SPEEK, and Zn&Sr‐SPEEK; F) Zn 2p high‐resolution spectra of Zn‐SPEEK and Zn&Sr‐SPEEK; G) Sr 3d high‐resolution spectra of Sr‐SPEEK and Zn&Sr‐SPEEK; H) contact angle and corresponding photos of droplets on various samples, *n* = 5.

The prepared products after hydrothermal treatment were characterized by scanning electron microscopy (SEM), transmission electron microscopy (TEM), and selected area electron diffraction (SAED). The SEM images (Figure S1A, Supporting Information) show the agglomerated small particles of ZnO and Sr(OH)_2_ in the form of nanocubes and short rod‐like nanoparticles, respectively. The high‐resolution TEM (HR‐TEM, **Figure** **1**A) images reveal that the interplanar spacings of 0.281 and 0.248 nm should be consistent with the (100) and (101) planes of the hexagonal wurtzite ZnO phase. The (100) and (110) crystal planes of ZnO are exhibited in the SAED pattern, further indicating that the sample is hexagonal phase. On the other one, the *d*‐spacing between two lattice fringes measured to ≈0.247 and 0.281 nm, which corresponds to the (102) and (021) planes of orthorhombic Sr(OH)_2_ phase, cross‐confirmed by the (123) and (021) crystal planes of Sr(OH)_2_ exhibited in the SAED pattern. The surface morphology of nutrient element (Zn and/or Sr) coatings on SPEEK substrates are illustrated in Figure 1B. The bare PEEK shows a flat surface. After sulfonation, SPEEK is altered to exhibit three‐dimensional (3D) hierarchical porous structures with highly interconnected open pores in size of 1.66 ± 0.59 µm (Figure S1B, Supporting Information). However, the 3D microporous structure was highly preserved after the hydrothermal treatment. ZnO nanocubes with a mean diameter of 0.46 ± 0.17 µm (Figure S1C, Supporting Information) are homogeneously distributed on both inner and outer surfaces of cavities in the Zn‐SPEEK samples. By contrast, plentiful Sr(OH)_2_ particles with sizes of 0.65 ± 0.18 µm (Figure S1D, Supporting Information) are uniformly distributing for the Sr‐SPEEK group. The coexistence of ZnO and Sr(OH)_2_ particles is expectedly observed on the porous surface of Zn&Sr‐SPEEK substrates after dual decoration. To cross‐check the results, the elemental mapping profiles (Figure S1E, Supporting Information) substantiate the existence of Zn and Sr in the coating. The specific surface area of the implant is enlarged by 3D hierarchical porous tomography, which significantly elevates the property for anchoring metal ions.

The crystal structure of the hydrothermal products was characterized by X‐ray diffraction (XRD). As shown in Figure 1C, several typical characteristic peaks of ZnO (PDF#89‐0511) at 2*θ* value of 31.78°, 36.26°, 47.55°, 56.61°, 66.40°, and 67.97° are well indexed to crystal planes of (100), (101), (102), (110), (200), (112), respectively. And in Figure 1D, several typical characteristic peaks of Sr(OH)_2_·H_2_O (PDF#77‐2336) at 2*θ* value of 26.53°, 31.78°, 36.34°, 40.45°, 45.92°, and 47.00° are well indexed to crystal planes of (002), (021), (102), (121), (031), (122), respectively. Hence, the modality of Zn and Sr elements in the self‐assembled coatings, ZnO and Sr(OH)_2_, has been demonstrated.

To further verify the results acquired from XRD, more proofs of chemical constitutes and states were supplied by X‐ray photoelectron spectroscopy (XPS). As illustrated in Figure 1E, the initial SPEEK merely exhibits prominent carbon and oxygen peaks as the main elements. Nevertheless, the signals of the Zn and Sr elements are detected in Zn&Sr‐SPEEK, further suggesting that the successful embellishment of Zn and Sr elements on the SPEEK surface. Furthermore, high‐resolution spectra of Zn 2p and Sr 3d for nutrient element modified SPEEK are provided in Figure 1F,G. As illustrated in Figure 1F, the peaks corresponding to Zn 2p1/2 and Zn 2p3/2 are singlets, without auxiliary binding energy peaks, symbolizing the divalent characteristic of the Zn ions (Zn(II)). Similarly, the divalent feature of Sr ions (Sr(II)) is confirmed from the high‐resolution XPS. Additionally, binding energies of O 1s on SPEEK (Figure S1F, Supporting Information) at 531.2, 532, and 533.3 eV are deconvoluted to the O_1_ (—O═C), O_2_ (—O═S), and O_3_ (—O—C). Two fresh oxygen XPS peaks arising between O_1_ (—O═C) and O_3_ (—O—C) components are indicative of O—Zn groups (O_4_) and O—Sr groups (O_5_), after successful self‐assembly of nutrient elements (Zn and Sr, Figure S1G, Supporting Information). Collectively, from these characterization results, we fully corroborate those nutrient elements (Zn and/or Sr) successfully decorated onto the SPEEK surface. Next, using the sessile drop method, water contact angle (WCA) was assessed to evaluate the surface hydrophilicity of samples (Figure 1H). As a comparison purpose, the WCA of PEEK is 107.67° ± 3.50°, indicating the hydrophobic feature of PEEK. Sulfonation treatment slightly decreases the WCA of the surface to a certain extent (94.03° ± 1.48°) but still with a hydrophobic surface. After modification with Zn and Sr elements, a significant decrease is observed, and the WCAs for Zn‐SPEEK, Sr‐SPEEK, and Zn&Sr‐SPEEEK are 85 ± 2.16°, 73.67 ± 2.32°, and 75.83 ± 2.95°, respectively. Previous literature has proved that a wettable surface can facilitate the initial adhesion of osteoblasts for subsequent osteogenic differentiation.^[^ ^37^ ^]^

It is extremely important to determine the liberation of nutrient elements (Zn and Sr) from the coatings for subsequent cell and bacteria responses. Their concentrations were tested using ICP through immersing samples in phosphate‐buffered saline (PBS, pH 7.4) at 37 °C over 7 d (Figure S1H,I, Supporting Information). Compared with Zn‐SPEEK, the release of zinc ions on Zn&Sr‐SPEEK is more moderate. Undergo a short burst‐like release to an initial peak concentration, Zn and Sr ions become mild during the following 7 d and reach a stable concentration. The cumulative release of Zn is 0.68 mg L^−1^ from Zn‐SPEEK, while Sr is 2.10 mg L^−1^ from Sr‐SPEEK. Besides, the levels of Zn and Sr ions delivered from Zn&Sr‐SPEEK samples are lower compared with that of Zn‐SPEEK and Sr‐SPEEK at each checkpoint. Zinc in the skeleton has been acknowledged to have an essential role in the formation and mineralization of bone. Also, it is identified that Zn^2+^ possesses multiple activities of inhibiting bacteria,^[^ ^32^ ^]^ including 1) charge transfer, 2) disruption of protons circulation and metabolism, and 3) protein denaturation. Furthermore, a proper supply of Zn^2+^ is vital to keep mitochondrial homeostasis,^[^ ^38^ ^]^ which is imperative for the differentiation and maturation of osteoblasts. While Sr^2+^ plays a dual‐acting role in bone reconstruction, inhibiting bone resorption and stimulating bone formation.^[^ ^39^ ^]^ We hypothesize that dual nutrient elements (Zn and Sr) can combine these benefits to maximize the diabetic osseointegration after spontaneously assembled on the surface of SPEEK.

### 2.2 Antibacterial Property

**Figure 2:**
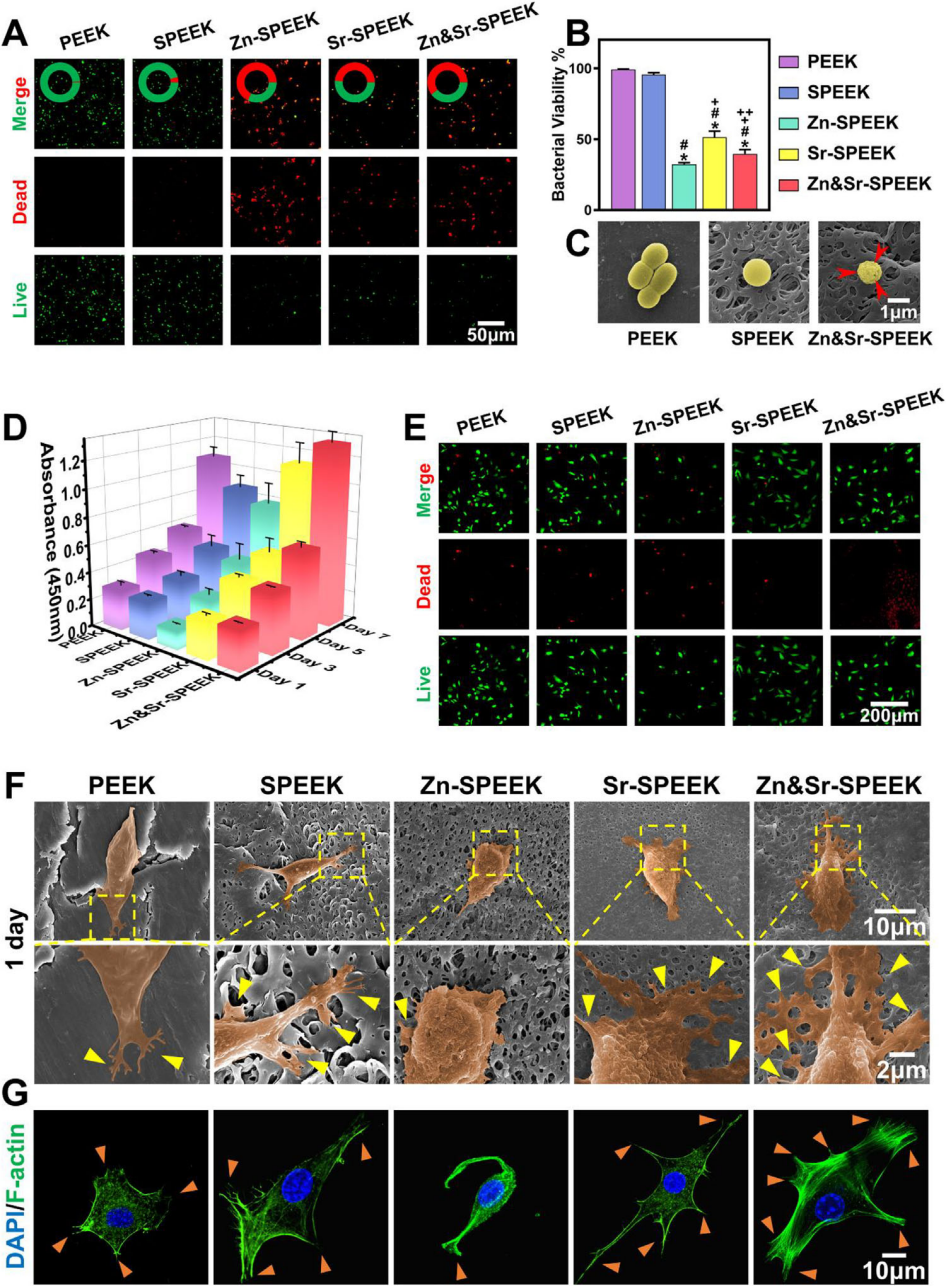
In vitro biocompatibility of diverse functionalized PEEK implants: A) Live/Dead fluorescent images of *S. aureus* on different PEEK substrates (live bacteria, green; dead bacteria, red), and B) their quantitative results, one‐way ANOVA and Tukey's post hoc test; *n* = 3; C) SEM morphologies of bacteria incubated with different samples (the red arrows indicate the shrinking membranes); D) CCK‐8 results of MC3T3‐E1 seeded on different substrates for 1, 3, 5, and 7 d, two‐way ANOVA and Tukey's post hoc test, *n* = 5; E) Calcein‐AM/PI double staining of cells on different substrates for 1 d (Calcein‐positive cells, green; PI‐positive cells, red); morphologies of cells cultured on various samples for 1 d captured by F) SEM (The MC3T3‐E1 cells are pseudocolored to orange for visual observation) and G) CLSM (the yellow and orange triangles represent the cellular pseudopodia). (*, #, +, and ++ represent *p* < 0.05 when compared with PEEK, SPEEK, Zn‐SPEEK, Sr‐SPEEK, respectively.)

The prerequisite step for the pathogenesis of infection is the colonization of bacteria to peri‐implant.^[^ ^40^ ^]^ It is effective for implantation's long‐term success that prevents bacterial attachment and proliferation on implants during the perioperatively.^[^ ^41^ ^]^ Thus, the antibacterial activity of the decorated SPEEK surfaces toward Gram‐positive *Staphylococcus aureus* (*S. aureus*), the typical bacteria responsible for implant complications of infection, was evaluated on the samples. The bacterial Live/Dead staining experiment was conducted. With the incorporation of nutrient elements, the density of the bacteria reduces, and the proportion of the dead bacteria increase dramatically, but no noticeable difference is found between SPEEK and PEEK (**Figure** **2**A,B). Therefore, the Zn and Sr ions delivered from samples can effectively inhibit the adhesion and growth of *S. aureus*. The morphology of *S. aureus* is presented in Figure 2C. It exhibits a relatively smooth and integrated surface in a uniform spherical shape on the original PEEK and SPEEK, while the rough surface with a lot of wrinkles in an irregular shape on the Zn&Sr‐SPEEK group, indicating that Zn&Sr‐SPEEK inhibits bacteria growth to some extent via contact killing mode.

### 2.3 In Vitro Biocompatibility of Zn&Sr‐SPEEK

Apart from antibacterial property, the cytocompatibility of a novel orthopedic coating is very crucial.^[^ ^42^ ^]^ The biocompatibility of Zn&Sr‐SPEEK was evaluated in simulated sustained hyperglycemic micromilieu (glucose, 25 × 10^−3^ m), including cell viability, initial adhesion activity, and spreading behavior. The cell counting kit 8 (CCK‐8) assay was applied to evaluate the proliferation of MC3T3‐E1 cells on samples for 1, 3, 5, and 7 d (Figure 2D). The cell viability on Zn‐SPEEK is statistically lower than that on other substrates on the first day, which might be attributed to the partially high concentration of released Zn^2+^. However, 3 d later, there is no remarkable difference between Zn‐SPEEK and SPEEK. The cell proliferation on Sr‐SPEEK and Zn&Sr‐SPEEK show a noticeable increase from SPEEK by day 5, probably on account of the sustained liberation of Sr^2+^ in the surroundings. Simultaneously, MC3T3‐E1 cells on Zn&Sr‐SPEEK exhibit the highest proliferation activity compared to those on Zn‐SPEEK and Sr‐SPEEK. It has been proved that Zn^2+^ ions show dose‐dependent toxicity toward mammalian cells,^[^ ^43^ ^]^ and appropriate concentration of Zn^2+^ (< 0.15 mg L^−1^) enables to facilitate the cell proliferation.^[^ ^44^ ^]^ In the liberation experiment, we can see from Figure S1H (Supporting Information) that the released concentration of Zn^2+^ ions for the Zn&Sr‐SPEEK group is about 0.1 mg L^−1^, which can significantly promote cell viability of MC3T3‐E1 cooperated with Sr^2+^ ions, ultimately resulting in the highest MC3T3‐E1 viability. Besides, after cultured for 24 h, the Live/Dead cells stained with the calcein acetoxymethyl ester (calcein‐AM, green fluorescence) and propidium iodide (PI, red fluorescence) were observed by a confocal laser scanning microscope (CLSM). As shown in Figure 2E, demonstrated by the presence of red fluorescence, Zn‐SPEEK shows a similar number of dead cells to the SPEEK and less cell colonization at the first 24 h. However, in the Zn&Sr‐SPEEK group, there are the fewest dead cells and most adherent live cells as indicated by green fluorescence in comparison with those on others. The aforementioned results are verified mutually in the works of cell viability in Figure 2D. The cell experiment results demonstrate that Zn&Sr‐SPEEK favors the proliferation of MC3T3‐E1, exhibits a prominent cytocompatibility.

The topography of MC3T3‐E1 osteoblasts cultivated on samples for 1 d was photographed by SEM displayed in Figure 2F. After 1 d, cells adhere to each substrate surface and spread generously with numerous filopodia anchored into the pores. Benefit from the 3D hierarchical porous structure and hydrophilicity, SPEEK‐based substrates exhibit better cell anchoring activity. An inferior cell adhesion with spindle‐shaped is found on SPEEK, indicating that the cells are reluctant to adhere to its surface. However, a more considerable spreading area of cells exhibits in Zn&Sr‐SPEEK, and the cytoplasm extended pseudopodia of filament on Zn&Sr‐SPEEK samples, indicating favorable adhering and spreading properties of osteoblasts on dual nutrient element coatings. According to the number and stretching of filopodia, cells on Zn&Sr‐SPEEK are distinctly tightest compared with those of others, while cells on Zn‐SPEEK appear to have shrunk. With the incubation time extended to 7 d (Figure S2, Supporting Information), MC3T3‐E1 cells are fully spread with direct cell‐to‐cell interactions on Zn&Sr‐SPEEK surface, while relatively dispersed and poorly spread on PEEK and Zn‐SPEEK, indicating better cytocompatibility of Zn&Sr‐SPEEK. The SEM results were further verified by CLSM images (Figure 2G), where the nucleus was stained with DAPI (blue) and the actin cytoskeleton with TRITC‐phalloidin (green). On Zn&Sr‐SPEEK, cells exhibit typical longitudinal shapes with radially organized actin fibers, whereas on SPEEK, they spread irregularly with nebulous and punctuated F‐actin filaments. These results illustrate that MC3T3‐E1 cells have optimal early adhesion and spreading on the Zn&Sr‐SPEEK in comparison with on the others, which may result from the co‐contribution of its hydrophilicity, proper surface roughness, and appropriate Zn^2+^ and Sr^2+^ liberation. After 3 d of incubation, cells grow into the 3D hierarchical topological structure confirmed by rotation of 3D reconstructions (Video S1, Supporting Information) from *Z*‐axis stacks of confocal images, which provides the profitable conditions for the excellent early osteogenic fixation.

### 2.4 Osteogenic Differentiation of Osteoblasts in Hyperglycemic Micromilieu

**Figure 3:**
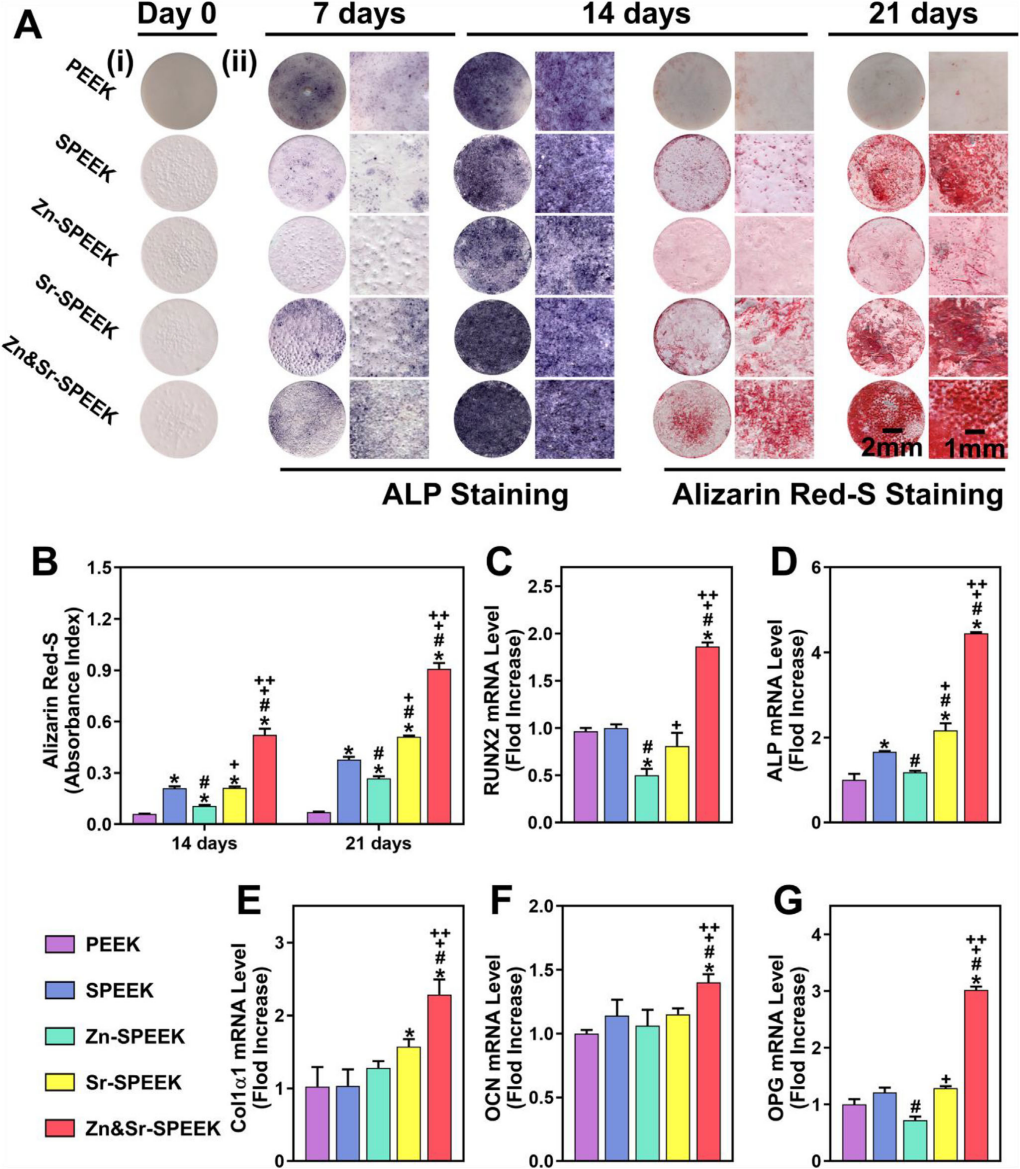
In vitro osteogenic potential in mimicked hyperglycemic microenvironment. A‐i) Photographs of different functionalized PEEK samples; ii) photographs of ALP and Alizarin Red S staining of MC3T3‐E1 cells on various substrates at each checkpoint. B) Quantification of ARS for mineral ECM, two‐way ANOVA and Tukey's post hoc test, *n* = 3; osteogenesis‐related genes relative expression of C) RUNX2, D) ALP, E) COL1*α*1, F) OCN, and G) OPG detected on day 7 by qRT‐PCR analysis, one‐way ANOVA and Tukey's post hoc test; *n* = 3. (*, #, +, and ++ represent *p* < 0.05 when compared with PEEK, SPEEK, Zn‐SPEEK, Sr‐SPEEK, respectively.)

As an early hallmark for osteogenic differentiation,^[^ ^45^ ^]^ the ALP staining was performed to detected ALP expression of MC3T3‐E1 cells cultivated on various substrates to differentiate whether the substrates possess the capacity to accelerate bone formation. To eliminate the interference of the color of samples, we first observed the substrate. Except for the PEEK group, there is almost no visual difference among the samples (**Figure** **3**A ‐i). Figure 3A ‐ii reveals that as time progress, ALP expression markedly increases in each group. As expected, ALP expression is highest in the Zn&Sr‐SPEEK group among others on both the 7th and 14th days, which can be attributed to the cells prefer to proliferate on Zn&Sr‐SPEEK substrates. Moreover, we find the darkest and densest blue staining in the Zn&Sr‐SPEEK group on the 14th day, implying the highest ALP expression. These results demonstrate that the considerable effects of Zn and Sr co‐modification on early osteogenesis.

Another critical biomarker of osteogenic differentiation is calcium nodules formation.^[^ ^46^ ^]^ Being an innate character of bone‐like structures, it plays an imperative role during the last stage of osteogenesis.^[^ ^47^ ^]^ After 14 and 21 d of incubation, Alizarin Red‐S (ARS) staining and semi‐quantitative analysis were performed concomitantly to evaluate the mineralization efficiency of the samples. Pristine PEEK displays few and scattered calcium nodes after 14 and 21 d of osteogenic induction. In comparison with the PEEK and SPEEK groups, Sr‐SPEEK and Zn&Sr‐SPEEK groups show significant augment in calcium nodules. Especially, the most intensive and extensive area of calcium nodules is found in the Zn&Sr‐SPEEK group, which is further validated by the quantitative analysis (Figure 3B). In the above experiment, Zn and Sr dual nutrient element coatings display effectiveness in facilitating extracellular matrix mineralization and calcium deposition.

To further probe the osteogenicity, the expression of osteogenesis‐related genes^[^ ^48^ ^]^ of osteoblasts encoding RUNX2, ALP, COL1*α*1, OCN, and OPG cultured on various samples were examined by quantitative real‐time polymerase chain reaction (qRT‐PCR) on day 7 (Figure 3C–G). Compared with the other groups, the osteoblasts cultured on the Zn&Sr‐SPEEK group exhibit the highest mRNA levels in terms of RUNX2, ALP, COL1*α*1, OCN, and OPG. These findings confirm that the osteogenic differentiation capacity is drastically ameliorated when the two nutrient elements work synergistically compared to use separately. Besides, when the Zn element is used alone, both cell and bacterial growth are inhibited (Figure 2A–E). After the Sr element is incorporated, the inhibitory effect on the cells is wholly relieved, while the antibacterial capability is retained. The result proves that the synergy of the two elements is satisfactory and efficient. It can acquire equilibrium between the osteogenic differentiation of cells and inhibition of the pathogenic bacterial growth during the perioperatively.

### 2.5 Mitochondrial Evaluations of MC3T3‐E1 Osteoblasts

Since mitochondrial dysfunction has been substantiated as one of the mechanisms of complications of DM,^[^ ^49^ ^]^ we were committed to exploring the mitochondrial dynamic and functional alterations in osteoblasts upon hyperglycemia through Zn and Sr mediation. MC3T3‐E1 cells, therefore, were cultivated in high glucose medium (33 × 10^−3^ m glucose) with different sample extracts to investigate the mitochondrial alterations in terms of mitochondrial fission and fusion (MFF), ROS production, and mitochondrial membrane potential (MMP).

#### 2.5.1 Mitochondrial Dynamics

**Figure 4:**
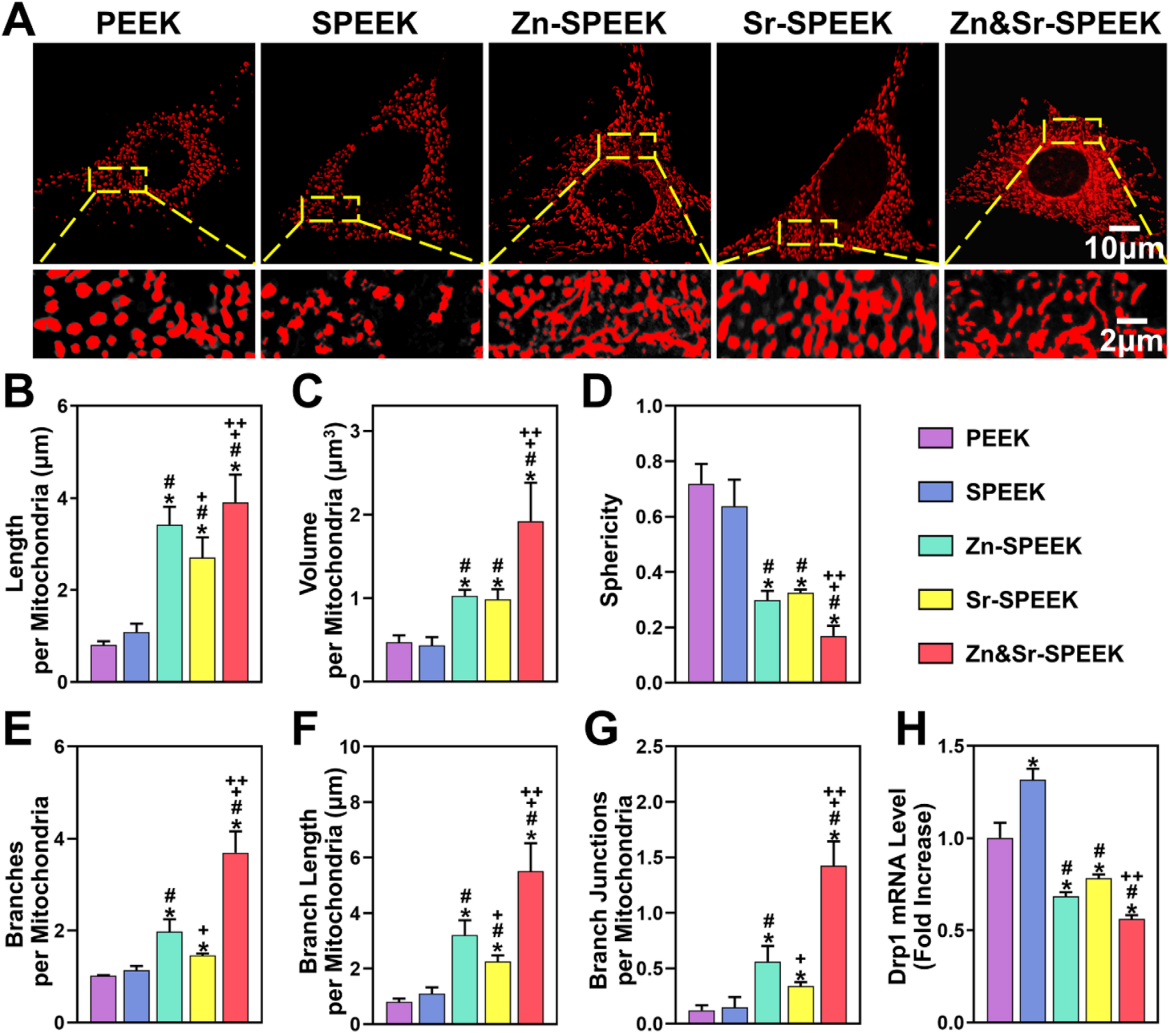
Mitochondrial dynamic evaluations of osteoblasts in mimicked hyperglycemic microenvironment. A) Representative fluorescent images of Mitotracker Red staining. Quantitative analysis of mitochondrial morphology and network connectivity including B) mitochondrial average length, C) volume, and D) sphericity, as well as E) mitochondrial average branches, F) branch length, and G) junctions, *n* = 5. H) Relative expression of Drp1 gene analyzed at 24 h by qRT‐PCR analysis, *n* = 3. (One‐way ANOVA and Tukey's post hoc test; *, #, +, and ++ represent *p* < 0.05 when compared with PEEK, SPEEK, Zn‐SPEEK, Sr‐SPEEK, respectively.)

Normal structure and morphology are essential for the function of mitochondria.^[^ ^50^ ^]^ Mitotracker Red staining was used to visualize ultrastructure for quantifying mitochondrial length, volume, and network appearance.^[^ ^51^ ^]^ Morphologically, mitochondria in the PEEK and SPEEK groups are shorter, rounder, and fragmented with less mitochondrial network continuity, which is reversed in the Zn&Sr‐SPEEK group with rod‐like and elongated mitochondria (**Figure** **4**A). Then, we carried out 3D reconstruction of mitochondria and quantified mitochondrial morphology and network appearance. The mitochondrial average length (Figure 4B), volume (Figure 4C), and sphericity (Figure 4D) reflect the mitochondrial morphology, and the mitochondrial average branches (Figure 4E), branch length (Figure 4F), and junctions (Figure 4G) reflect the mitochondrial network complexity. When the Zn and Sr elements are used alone, mitochondrial morphology has a few improvements compared with that in PEEK and SPEEK groups. These results also declare that Zn has a positive effect on both mitochondrial morphology and network structure, while Sr seems to mainly maintain the stability of morphology. Therefore, the synergistic effect of nutrient elements (Zn and/or Sr) boosts the mitochondrial dynamics regulation ability.

Drp1 executes the fission process, and its overexpression brings about excessive mitochondrial fragmentation.^[^ ^52^ ^]^ Given that the equilibrium of MFF is imperative for the conservation of normal mitochondrial ultrastructure.^[^ ^53^ ^]^ We next sought to ascertain whether the Drp1 expression level was altered in MC3T3‐E1 cells of indicated groups. As shown in Figure 4H, Zn&Sr‐SPEEK considerably decreases Drp1 expression by 0.56‐fold compared to the PEEK group. Accompanied with the results of Mitotracker Red staining, these findings indicate that Zn and Sr elements have a corporative inhibitory effect on excessive mitochondrial fission and its concomitant impaired mitochondrial function, stemming from high sugar.

#### 2.5.2 Mitochondrial Functions

**Figure 5:**
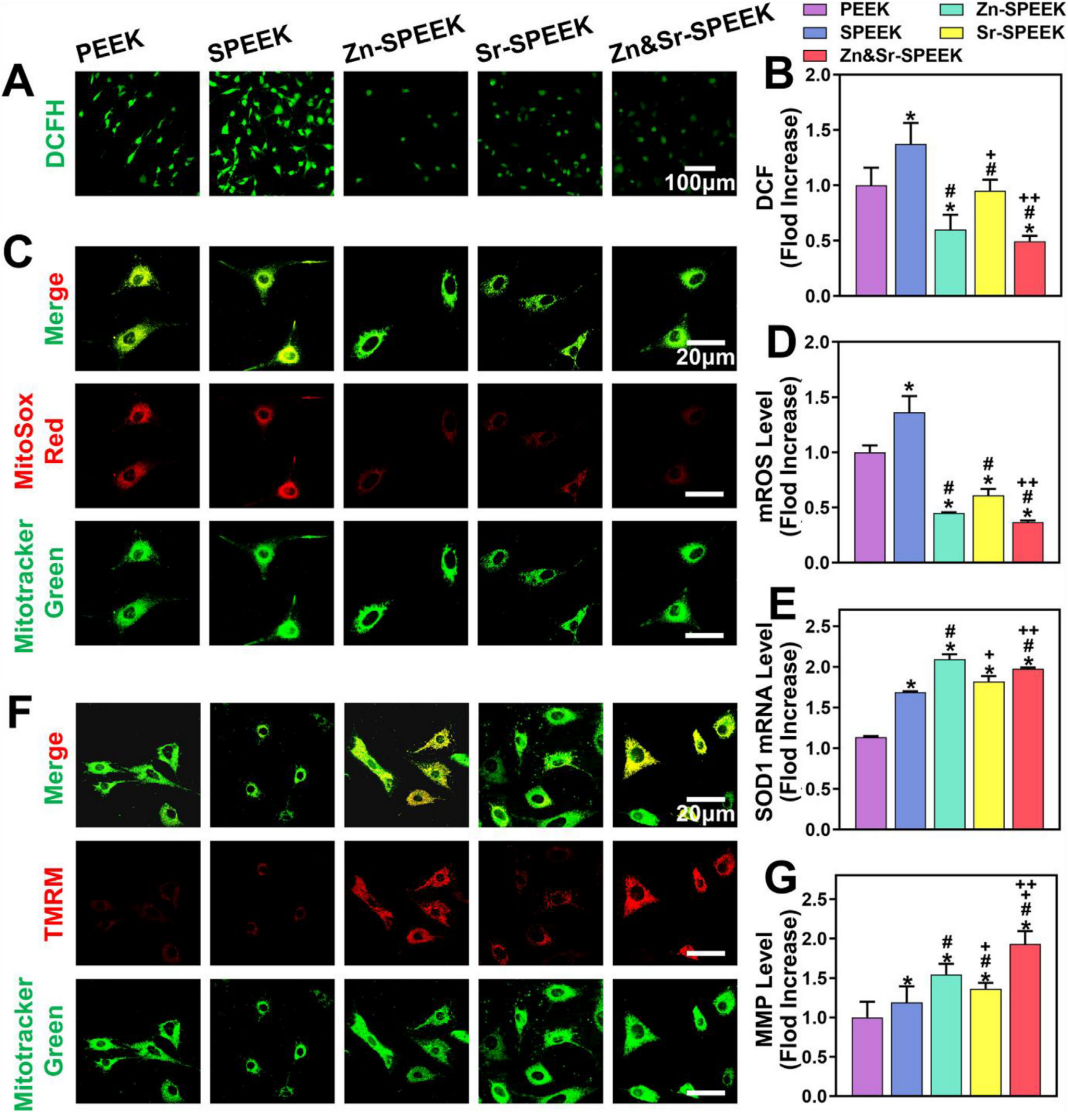
Mitochondrial function assessments of osteoblasts in mimicked hyperglycemic microenvironment: A,B) representative fluorescent images and quantification of DCFH staining for intracellular ROS, *n* = 5; C,D) representative fluorescent images and quantification of MitoSOX staining for mitochondrial ROS, *n* = 5; E) relative expression of SOD1 gene detected at 24 h by qRT‐PCR analysis, *n* = 3; F,G) representative fluorescent images and quantification of TMRM staining for mitochondrial membrane potential, *n* = 5. (One‐way ANOVA and Tukey's post hoc test; *, #, +, and ++ represent *p* < 0.05 when compared with PEEK, SPEEK, Zn‐SPEEK, Sr‐SPEEK, respectively.)

Oxidative stress is an indispensable part of the physiological bone‐healing process, which resists pathogens and protects the body's homeostasis.^[^ ^54^ ^]^ Synchronously, excessive ROS production mediated by DM impairs bone regeneration and compromises the osseointegration of an implant.^[^ ^55^ ^]^ Although the organism has a self‐antioxidant defense system and metabolizes redox products physiologically, it has insufficient capacity to correct and reverse the imbalance. Thus, we assessed the intracellular ROS levels and antioxidant activities of osteoblasts cultured on various samples in the hyperglycemic micromilieu. The intracellular ROS was visually labeled by the fluorescent probe 2′,7′‐dichlorofluorescein diacetate (DCFH‐DA) to indicate the in vitro ROS‐scavenging properties of Zn&Sr‐SPEEK implants (**Figure** **5**A). Zn&Sr‐SPEEK samples exhibit the lowest fluorescence intensity (Figure 5B), thanks to the synergy of Zn and Sr elements. Simultaneously, the expression of Copper‐Zn Superoxide Dismutase (SOD1) was detected, and qRT‐PCR results show that Zn&Sr‐SPEEK significantly augments the SOD1 level compared with the PEEK group (Figure 5E). Considering mitochondria as the primary source of ROS generation, a highly selective fluorescent dye (MitoSox Red) was employed to determine mitochondrial ROS levels further. As predicted, the Zn&Sr‐SPEEK exhibits a drastically scavenging ability on mitochondrial ROS accumulation with the mean fluorescence intensity of MitoSox Red staining reduced to 0.37‐fold compared to the PEEK group (Figure 5C,D). The above results demonstrate that Zn&Sr‐SPEEK implants resist to diabetes‐mediated oxidative stress of osteoblasts through scavenging of ROS or enhancement of intracellular antioxidant defenses.

It is well‐known that excessive mitochondrial fission incites ROS accumulation.^[^ ^56^ ^]^ Conversely, superfluous ROS has been demonstrated to induce mitochondrial fragmentation, amplifying oxidative stress via the vicious cycle termed “ROS‐induced ROS release” and eventually results in mitochondria dysfunction.^[^ ^57^ ^]^ To assess the functional state of mitochondria, the MMP, a momentous marker of mitochondrial function,^[^ ^58^ ^]^ loss of which may purport the changes of mitochondrial membrane permeability,^[^ ^59^ ^]^ was measured by tetramethylrhodamine methyl ester (TMRM) staining (Figure 5F). It is observed that a slight increase in MMP when the Zn and Sr elements are used alone. As expected, the synergistic effect maximized the ability to resurge the MMP (Figure 5G). The quantitative flow cytometry analysis also confirmed the effectiveness of Zn&Sr‐SPEEK on preventing ROS damage and MMP resurgence (Figure S3, Supporting Information). Taken together, these results indicate that Zn&Sr‐SPEEK has a robust ability to recuperate hyperglycemia‐induced mitochondrial dynamic and functional alterations by means of Drp1 gene down‐regulation, MMP resurgence, and reactive ROS elimination (Scheme 1C). Thereby, Drp1 inhibition may be an indirect downstream factor accounting for the recovery of Zn&Sr‐SPEEK osteogenic function in hyperglycemia.

### 2.6 In Vivo Diabetic Rat Model Verification Experiments

**Figure 6:**
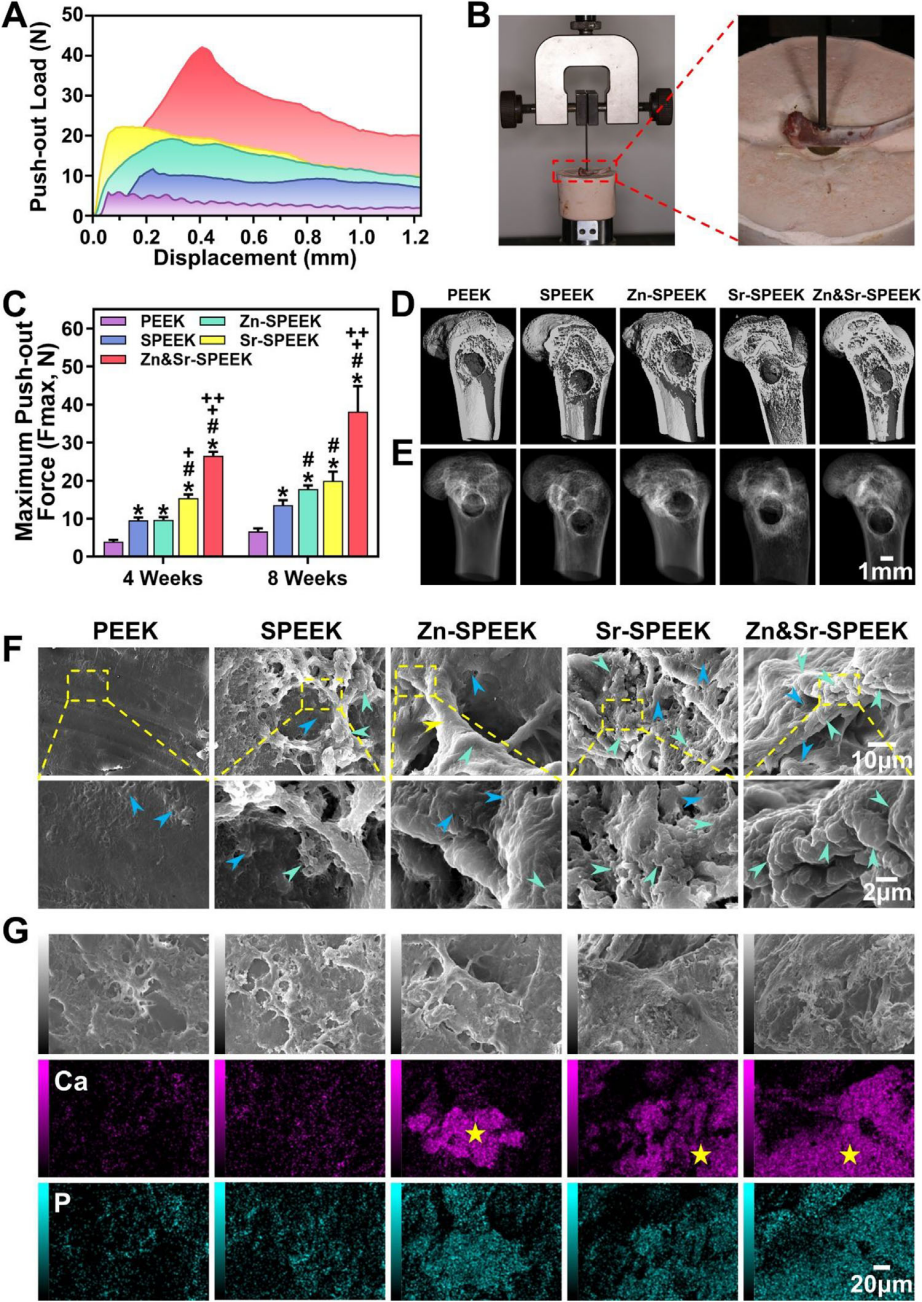
In vivo osseointegration study around implants in diabetic rat model: A) load‐displacement curves and C) the average push‐out forces of the implants at 4 and 8 weeks; B) schematic diagram of push‐out experiment; D) micro‐CT reconstruction and E) X‐ray images of the sagittal plane at 8 weeks postoperatively; F) the bone attachment and growth onto the implant surfaces at 8 weeks observed by SEM. Green arrows indicate the calcified tissues, blue arrows point to the collagen, and yellow arrows indicate the microvascular. G) Corresponding SEM cross‐sectional and EDS‐mapping images at 8 weeks postoperatively. Yellow asterisk represents the calcified tissues. (Two‐way ANOVA and Tukey's post hoc test were used, *n* = 4; *, #, +, and ++ represent *p* < 0.05 when compared with PEEK, SPEEK, Zn‐SPEEK, Sr‐SPEEK, respectively.)

Encouraged by the in vitro cell experiments, these results spur us to investigate the effect of nutrient element coatings on implant osseointegration in the diabetic state. Hence, we established a femoral/tibia bone defect model on the diabetic rat (Scheme 1B) to confirm the in vivo osseointegration of decorated porous PEEK. Various implants were inserted bilaterally into the femurs and tibias of 40 rats. The rats were sacrificed humanely at 4 and 8 weeks postoperatively and harvest the tibias for biomechanical analysis and the femurs for histomorphometric and immunohistochemistry analysis. From 3D microcomputed tomography (micro‐CT) reconstruction (**Figure** **6**D and Figure S4A, Supporting Information) and X‐ray images of the sagittal planes (Figure 6E and Figure S4B, Supporting Information), implants are integrated with the surrounding bone tissues obviously without complications such as displacement and infection or inflammation after implantation. Desirable implant function requires secure fixation with the surrounding bone. Therefore, to investigate the bone‐implant contact strength of PEEK implants, biomechanical testing was conducted after implantation for 4 and 8 weeks using a computer‐controlled universal testing machine (Figure 6B). Figure 6A,C displays the representative load‐displacement curves and the corresponding average maximum push‐out force (Fmax) of implants. With time prolongation, osseointegration strength increases. Pristine PEEK has inferior bonding strength with the host bone due to the smooth and hydrophobic surface, regardless of 4 and 8 weeks, and the Fmax values are 3.93 ± 0.37 and 6.23 ± 0.40 N, respectively. With porous structure, the Fmax of SPEEK is increased to 9.53 ± 0.60 (4 weeks) and 13.17 ± 1.04 N (8 weeks). After further hydrothermal treatment and nutrient element modification, however, the Fmax of Zn&Sr‐SPEEK is improved sharply to 26.57 ± 0.82 and 38.13 ± 5.47 N at 4 and 8 weeks, respectively, which are much higher than those of SPEEK, Zn‐SPEEK, and Sr‐SPEEK. Therefore, the promoted osteogenesis effect is co‐contributed by the presence of Zn^2+^/Sr^2+^ and surface topology/roughness.

We further used SEM to observe the newborn tissues attached to the implants separated from the pushed‐out tibia bone (Figure 6F). According to the SEM analysis, the regenerated bone tissue has permeated the implants indicated by some micropores filling with the new bone tissues. Noteworthily, scratch surfaces are visible on PEEK implant yet, suggesting less bone tissue covering the surface and explaining why PEEK is easier to be pushed out. The bone creation surrounding the Sr‐SPEEK and Zn&Sr‐SPEEK implants are drastically more extensive than SPEEK at 4 weeks (Figure S5, Supporting Information). With time extending to 8 weeks, Zn&Sr‐SPEEK is almost wrapped with more regular and denser newly formed bone tissues, supporting the detected increased Fmax value. As for the energy‐dispersive X‐ray spectrometry (EDS) mapping (Figure 6G), the results corroborate that a large amount of calcium and phosphorus (newly developed calcified matrix) are deposited on the surface of the implant at 8 weeks, especially on Zn&Sr‐SPEEK. The above data disclose that after the nutrient elements (Zn and Sr) decoration, the implants can co‐trigger bone remolding and enhance mechanical anchoring of the implant, thereby ensuring long‐term osteogenic fixation.

**Figure 7:**
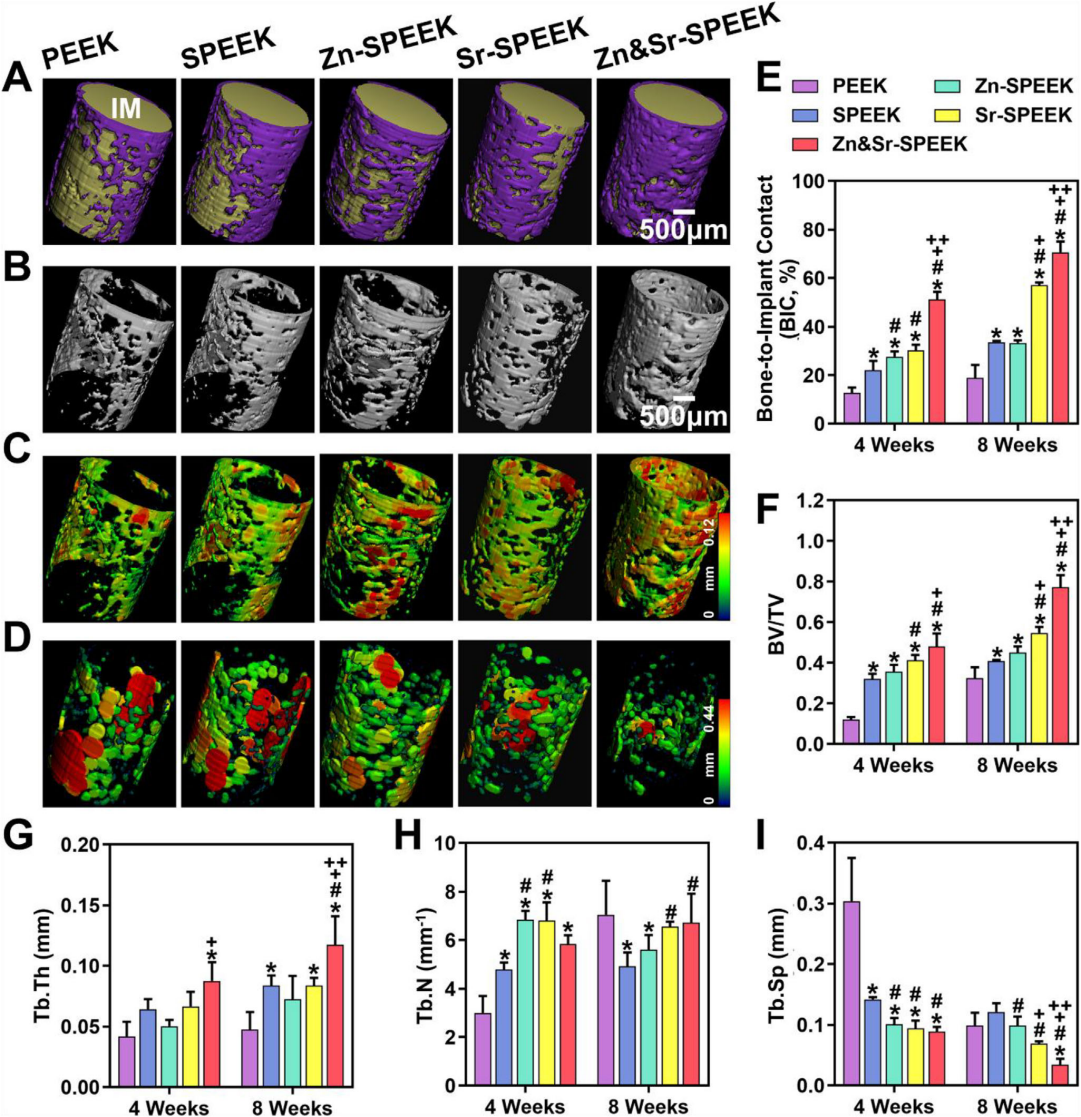
The quality and quantity of newly regenerated bone tissues surrounding the implants: the 3D reconstructed micro‐CT images of newborn bone A) with implants and B) without implants at 8 weeks, as well as E) the quantification of the bone‐implant contact value (implant (IM), yellow; new bone, purple). Representative images of C) Tb.Th and D) Tb.Sp at 8 weeks. Quantitative analysis of Micro‐CT data including F) BV/TV, G) Tb.Th, H) Tb.N, and I) Tb.Sp. (Two‐way ANOVA and Tukey's post hoc test were used; *n* = 4; *, #, +, and ++ represent *p* < 0.05 when compared with PEEK, SPEEK, Zn‐SPEEK, Sr‐SPEEK, respectively.)

The regenerated bone structure around the implants was reconstructed and analyzed at 4 and 8 weeks after implantation through micro‐CT. The 200 µm ring area extending from the implant periphery was included in morphologic and morphometric analyses to recognize the bone‐implant interface better. Due to the natural radiolucency of PEEK materials, it is more intuitive to assess the variations in complex bone formation around the implant. Visually, the reconstructed micro‐CT images exhibit new‐formed thin bone layers surrounding the implants in cancellous bone for 8 weeks postoperatively. The larger bone volume (**Figure** **7**A) and the higher bone‐implant contact rate (Figure 7E) around the Zn&Sr‐SPEEK implants compared with those around Sr‐SPEEK and Zn‐SPEEK group determined from qualitative and quantitative analysis (*p* < 0.05), suggesting that Zn&Sr‐SPEEK has the strongest in vivo osteogenicity ability at the macro level. The quantitative micro‐CT analysis coupled with corresponding images of new trabecular bone exhibits that the bone volume/tissue volume (BV/TV, Figure 7B,F) and trabecular bone thickness (Tb.Th, Figure 7C,G) in the Zn&Sr‐SPEEK implant are evidently highest among all groups (*p* < 0.05). Besides, a prominent decrease in trabecular separation (Tb.Sp, Figure 7D,I), defined as the distance between adjacent trabecula, is discovered from the Zn&Sr‐SPEEK group (*p* < 0.05), though there is no noteworthy difference in the trabecular bone number (Tb.N, Figure 7H) among the groups. Simultaneously, Zn&Sr‐SPEEK is embraced by high‐quality newborn bone tissues at 4 weeks (Figure S6, Supporting Information), which disclose the better early osteogenesis of Zn&Sr‐SPEEK. The preceding analyses demonstrate that the new‐formed bone surrounding the Zn&Sr‐SPEEK implants are distinctly better than those of the other groups in morphology and morphometry. And taking the time dimension into account, we found that compared with SPEEK, nutrient elements‐modified SPEEK exhibited increased bone quality over time. It demonstrates that the modified, especially double‐nutrient elements modified SPEEK, possess not only superior early osteogenesis but also long‐term bone regeneration.

In vivo experiments also indicate that Sr‐SPEEK and Zn&Sr‐SPEEK integrate better with host bone and induce prominent bone regeneration. Besides, according to micro‐CT analysis, Sr‐SPEEK and Zn&Sr‐SPEEK are dramatically better than SPEEK in bone histomorphometry, demonstrating that Sr drastically improves the osteogenesis and osteoinduction of SPEEK implants, especially in diabetic model. The stable liberation of Sr ions could promote osteoblastic proliferation in vitro, and expedite bone formation and osseointegration in vivo. However, the osteoinductive mechanism of strontium remains unknown. On the other hand, the quality of bone regeneration is further heightened by the modification of Zn,^[^ ^44^ ^]^ which significantly recuperates hyperglycemia‐induced mitochondrial dynamic and functional alterations and is reported to have better antibacterial activity. Base on the in vitro and in vivo results, the co‐functionalization of Sr and Zn enables to accelerate the early osteogenesis with satisfactory osteoinduction and harvest better in vivo bone remolding with desirable osteoconduction, thus realizing safe and effective osteogenic fixation.

**Figure 8:**
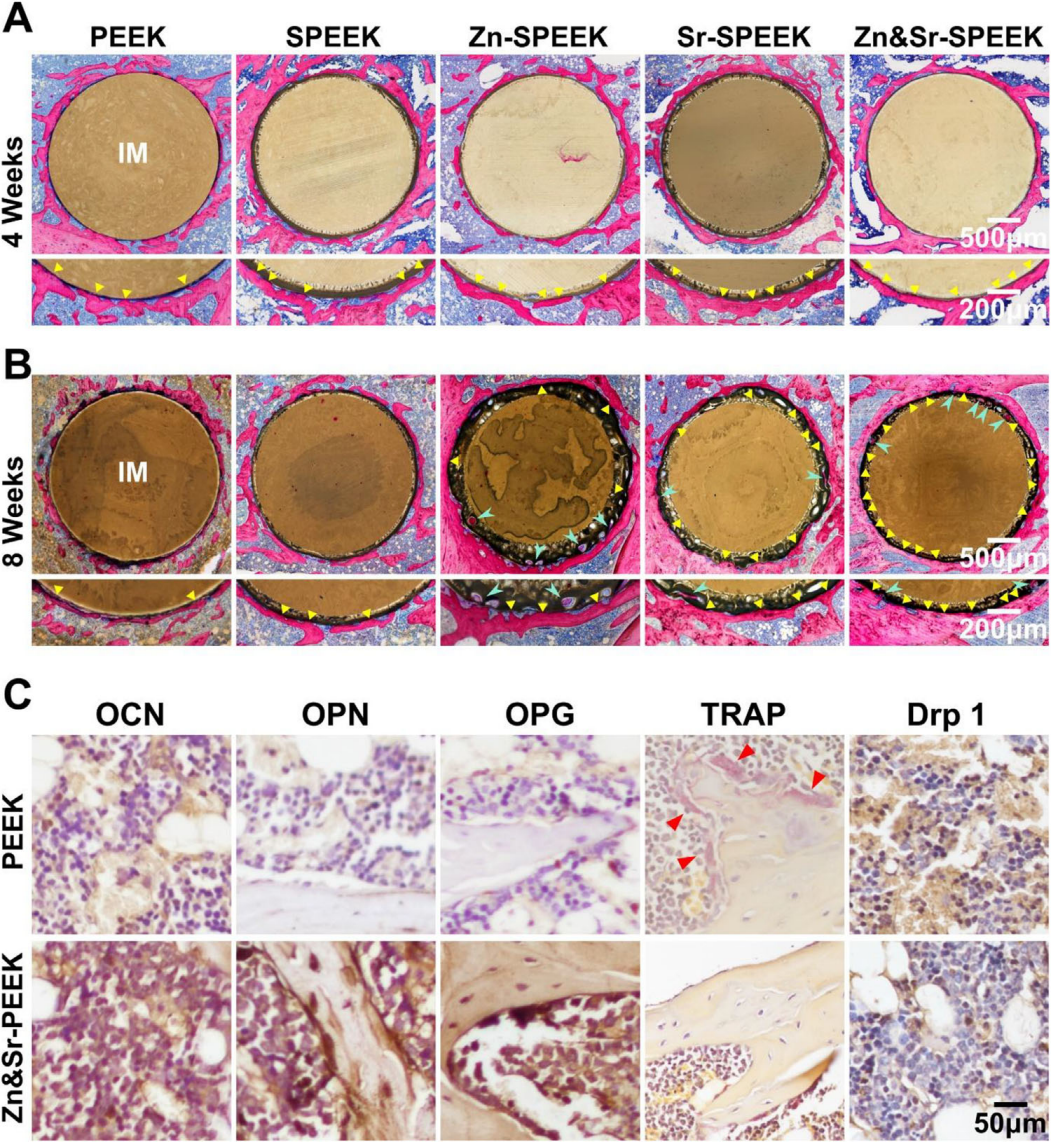
Histomorphometric and immunohistochemistry analysis: toluidine blue‐fuchsine staining at A) 4 and B) 8 weeks (yellow triangles reveal new bone in direct contact with the implant; green arrows point to new bone ingrowth into the implant); C) immunohistochemistry staining of OCN, OPN, OPG, TRAP, and Drp1 proteins around the implants at 8 weeks (red arrows indicate the osteoclasts).

In order to further analyze the regenerated bone and osseointegration, histological staining was operated in our study. In **Figure** **8**A,B, a thin neotissue layer around the implant is displayed through the toluidine blue‐fuchsine staining. More uniform and continuous new bones are found and tightly connected with the nutrient elements modified implants, especially for Zn&Sr‐SPEEK implants. The amount and maturity of new bone contacted with the implant are more satisfactory at 8 weeks for all groups. At 8 weeks, the newborn bone is tightly integrated on the surface of Zn&Sr‐SPEEK implants and extends along with the implant interface. And a prominent bone ingrowth into the inner pores can be observed clearly, showing good osteogenic fixation ability of Zn&Sr‐SPEEK implants.

To further corroborate the role of Zn and Sr elements in promoting osseointegration. Immunohistochemical staining was utilized to assess the activity of osteogenesis‐associated proteins in the peri‐implant region. We analyzed the positive labeling for osteopontin (OPN), osteocalcin (OCN), and osteoprotegerin (OPG) (Figure 8C). OCN, a bone‐specific matrix protein related to mineralization, is secreted by osteoblasts. OPN is an intermediate stage marker of osteogenic differentiation, connected with the synthesis of the organic matrix. OPG, a bone regeneration protein concerned with bone reconstruction, is a bone remodeling marker. Compared to those in the PEEK group, OCN, OPN, and OPG proteins are clearly upregulated in the Zn&Sr‐SPEEK group, which indicates that the implant possesses a remarkable ability to promote bone formation. Interestingly, more osteoclasts are found on the periphery of the new bone tissues on the surface of PEEK due to the diabetic microenvironment (Figure 8C). As a result of the advanced glycation end product in hyperglycemia, exciting cellular tissue destruction functions further promote the formation and activation of osteoclasts. The effect of Zn&Sr‐SPEEK during osseointegration is comprehensively appraised by histological and immunohistochemical analysis of bone sections. In the Zn&Sr‐SPEEK surface, we observe not only more new bone and tightly integration but also stronger early osteogenesis‐related protein expression, resulting in appealing osseointegration.

Since alterations in mitochondrial dynamics and Drp1 gene expression have been observed in in vitro experiments, it is worth confirming the alterations in vivo. As illustrated in Figure 8C, compared with the PEEK group, fewer positive areas are found around the Zn&Sr‐SPEEK group, which is in accordance with the in vitro results that benefit the mitochondrial dynamic equilibrium around diabetic implants. These results further confirm that Drp1 gene down‐regulation may be an indirect downstream factor for nutrient elements (Zn & Sr) to rescue hyperglycemia‐induced mitochondrial dynamic and functional alterations, thereby promoting diabetic osseointegration. Previous studies have used Cu‐containing scaffolds to achieve simultaneous bactericidal and bone formation‐promoting properties.^[^ ^60^ ^]^ However, it is difficult for a single element to balance the two abilities. In the current work, we decorate Zn and Sr elements onto orthopedic implants. Apart from possessing antibacterial properties, our nutrient elements decorated implants enable to significantly boost diabetic bone formation and osseointegration under the hyperglycemic microenvironment through steering mitochondrial dynamics, compared with these reported Cu‐containing scaffolds.

**Scheme 2:**
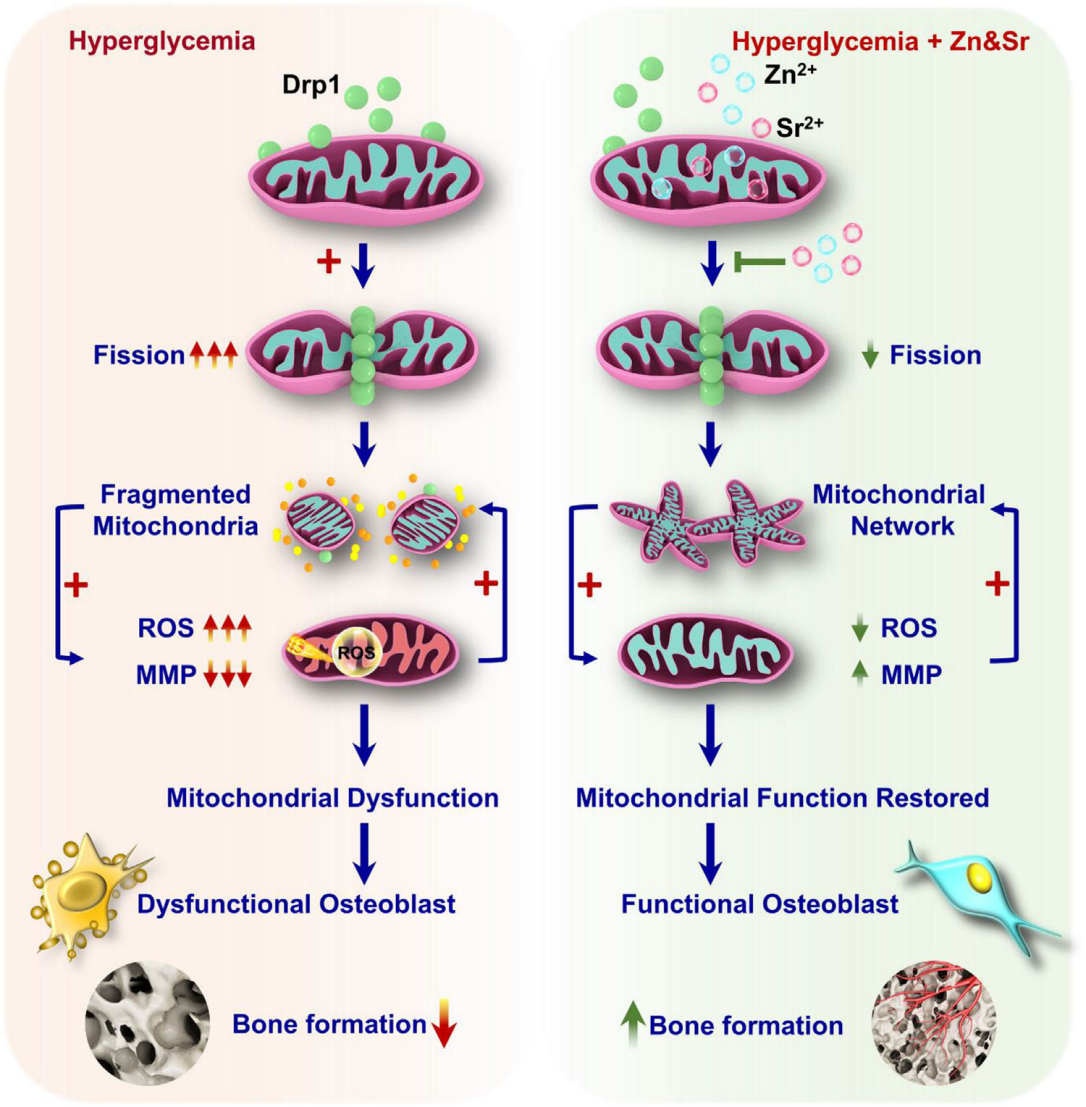
Schematic diagram depicting hyperglycemia‐induced osteoblast damage and the role of Zn&Sr elements in recuperating the functions of osteoblast via reducing Drp1‐related mitochondrial fission.

According to the above analysis, more newborn bone tissues around the Zn&Sr‐SPEEK were revealed in both micro‐CT and undecalcified section staining. Enhanced expression of OCN, OPN, and OPG proteins indicates the potential of dual nutrient elements (Zn & Sr) in the enhancement of osteogenic differentiation and bone formation, and the synergy effects may be another reason for the superior osteogenesis in the Zn&Sr‐SPEEK group. Most importantly, the liberation of nutrient elements (Zn and Sr) down‐regulates Drp1 overexpression caused by hyperglycemia and rescue mitochondrial dynamic equilibrium, which subsequently rebuilds the homeostasis of osteoblasts and establishes a preferable osteogenic microenvironment^[^ ^61^ ^]^ for osseointegration of an implant (**Scheme** **2**).

## 3 Conclusion

Herein, we successfully synthesized a Zn and Sr co‐decorated PEEK orthopedic implant through a facile hydrothermal process and investigated the direct relationship between mitochondrial dynamics and diabetic osteogenicity for the first time. The engineered dual nutrient elements decorated implants possessed excellent antibacterial capability toward pathogenic *S. aureus* and exerted superior cytocompatibility. In vitro results of hyperglycemic stress model demonstrated that benefitting from the collaborative delivery of Zn and Sr ions, Zn&Sr‐SPEEK implants enabled to inhibit the overexpression of Drp1 gene and decline the expression of ROS of cells, as well as recuperate mitochondrial membrane potential, ultimately contributing to regulating mitochondrial dynamics and functions upon hyperglycemic micromilieu. Moreover, enhanced osteogenic differentiation of osteoblasts under hyperglycemia and boosted in vivo osseointegration in diabetic rat bone defect models were found in Zn&Sr‐SPEEK implants. Accordingly, our work provides a new mitochondria‐target orthopedic implant that can steer mitochondrial dynamics to enhance osteogenesis, which broadens the potent application of PEEK biomaterials in diabetes therapy.

## 4 Experimental Section

### Fabrication of Nutritional Elements Decorated PEEK Implants

Biomedical grade PEEK disks (Φ8.5 × 2 mm^3^) were provided from Thornton Cleveleys (UK) and cleaned in acetone, ethanol, and ultrapure water with ultrasound in sequence. The samples were immersed into sulfuric acid (H_2_SO_4_, 98 wt%) with sonication for 5 min at room temperature to attain a 3D porous structure. Then, the residues of H_2_SO_4_ were removed by ultrasonic waves within in acetone and ultrapure water repeatedly. After that, the Zn‐SPEEK samples were synthesized via a hydrothermal method heated at 90 °C for 2 h with a mixed suspension containing 0.5 mol L^−1^ Zn(NO_3_)_2_·6H_2_O (Chengdu KeLong Reagent, China) and 0.5 mol L^−1^ hexamethylenetetramine (Chengdu KeLong Reagent) aqueous solution. Afterward, Zn‐SPEEK and SPEEK samples were infiltrated in 0.08 mol L^−1^ Sr(OH)_2_·8H_2_O (Chengdu KeLong Reagent) aqueous solution, processed by another hydrothermal reaction at 120 °C for 4 h to harvest Zn&Sr‐SPEEK and Sr‐SPEEK, respectively.

### Materials Characterization of Samples

The surface topography of the substrates was analyzed by a field‐emission SEM (FE‐SEM; JSM‐7500 F, JEOL, Japan). Elemental mapping was realized by EDS equipped on SEM machine. TEM (Tecnai G2 F20 S‐TWIN, FEI, USA), SEAD (taken in the TEM mode), and XRD (LabX, Shimadzu) were utilized to investigate the phase structures of the samples made by hydrothermal reaction. The elemental composition and chemical state of SPEEK surfaces were analyzed by XPS (AXIS Supra, Kratos, USA). The wettability of various substrates was determined by the WCA apparatus (Zhongchen Digital Technic Apparatus Co., Shanghai). Then the inductively coupled plasma atomic emission spectroscopy (ICP‐AES, 5100 SVDV, USA) was employed to evaluate the release of Zn and Sr ions in the PBS solution.

### Antibacterial Assessment

Live/Dead BacLight Bacterial Viability Kit (Invitrogen, USA) was used to investigate the antibacterial property of samples. Briefly, *S. aureus* suspension was inoculated onto the samples and cultured overnight. Then, bacteria on samples were dyed with a 1:1 mixture of SYTO 9 and PI for 15 min in the darkness and observed through CLSM. After 1 d of culture on substrates, *S. aureus* was fixed with 2.5% glutaraldehyde overnight. After that, the *S. aureus* with samples were dehydrated in gradient ethanol (30, 50, 75, 85, 95, 100%) for 15 min sequentially. Finally, bacteria were dried through critical point drying, sputtered with gold, and observed by FE‐SEM.

### Cytocompatibility Evaluation

Osteoblast precursor cells from Mouse, MC3T3‐E1, were generously offered by the State Key Laboratory of Oral Diseases, West China Hospital of Stomatology. The cells were cultured in alpha minimal essential medium (*α*‐MEM; Gibco, USA) supplemented with 10% fetal bovine serum (FBS, Gibco) and 1% antibiotic solution (penicillin/streptomycin, Gibco) at 37 °C in a humidified incubator with 5% CO_2_ atmosphere. They were passaged by trypsinization after 80% confluence, and the growth media were changed every 2–3 d.

### Cell Viability Assay

MC3T3‐E1 cells (1 × 10^4^ cells per well) were seeded onto each sample in the 48‐well plate with 25 × 10^−3^ m high‐glucose (HG) Dulbecco's modified eagle media (DMEM; Gibco) to mimic a sustained hyperglycemia microenvironment. Cell viability was performed when cells were cultured in HG media for 1, 3, 5, and 7 d using a CCK‐8 (Dojindo, Japan). At each checkpoint, the media was replaced with a 300µL mixed solution (1:10 of CCK‐8 reagent to media) and was co‐incubated at 37 °C for 1.5 h. After shaken for 10 s gently, 100 µL of the supernatants were transferred into a new 96‐well plate separately, and the absorbance was measured using a microplate reader (Thermo Scientific, USA) at 450 nm.

In addition, Live/Dead staining was detected to access the cell viability on substrates qualitatively. Briefly, MC3T3‐E1 cells were inoculated onto each sample in a 48‐well plate for 24 h. Then, 300 µL of 8 × 10^−6^ m PI and 2 × 10^−6^ m calcein‐AM in PBS was incorporated into each sample and incubated for 0.5 h. After rinsed with PBS, cells on samples were captured using CLSM.

### Cell Morphology and Immunofluorescence Staining

After 1 and 7 d cultured, the cells on substrates were observed by FE‐SEM through the same processing procedure as *S. aureus* in *Antibacterial Assessment*.

Besides, TRITC‐phalloidin (Solarbio Corporation, Beijing, China) was used to mark F‐actin to observe the cytoskeleton. After 1 d of incubation, the cells were fixed with 4% PFA for 10 min and permeabilized with 0.1% of Triton X‐100 (Sigma, USA) in PBS for 15 min. Subsequently, the cell cytoskeleton was stained by TRITC‐phalloidin and the nuclei by and 4′,6‐diamidino‐2‐phenylindole (DAPI; Solarbio Corporation). Fluorescent imaging of cells was detected under CLSM.

### Osteogenic Potential Evaluation

MC3T3‐E1 cells (1 × 10^4^ cells per well) were seeded on various substrates in the 48‐well plate. After 90% of confluence, the growth media was replaced with osteogenic inducing media consisting of DMEM supplemented with 100 × 10^−9^ m dexamethasone (Sigma), 50 µg mL^−1^ ascorbic acid (Sigma), and 10 × 10^−3^ m *β*‐glycerophosphate (Sigma). The cells undergo osteogenic differentiation under a hyperglycemia condition and the media were updated every 2 d.

### ALP Staining

ALP staining was conducted according to the below protocol. 7, 14 d after osteogenic induction, samples were rinsed with PBS, fixed by 4% PFA for 30 min at 4 °C, and then stained by a BCIP/NBT ALP color development kit (Beyotime, China) followed the manufacture's protocol. Finally, cells were washed with ultra‐pure water, and dyed images were captured by an Epson Perfection V370 Photo Scanner (Japan).

### ARS Staining

After 14 and 21 d of osteogenic induction, samples were washed with PBS and fixed in 95% ethanol for 10 min at ambient temperature. Afterward, samples were stained with 0.1% Alizarin Red (Sigma) dissolved in Tris‐HCl (pH 8.3) for 30 min, and mineralized nodules will be stained to dark red. Images were captured using Epson Perfection V370 Photo Scanner. Quantitative analysis of ARS was attained by dissolving the nodules with 10% cetylpyridinium chloride (Chengdu KeLong Reagent) overnight. The quantitative result was measured by absorbance at 562 nm with a microplate reader (Thermo Scientific).

### qRT‐PCR

Total RNA was extracted using the RNA Extraction Kit (TAKARA, Japan) following the manufacturer's instruction and the RNA quality confirmed by NanoDrop (Thermo Scientific). After cDNA was synthesized with the PrimeScriptRT reagent Kit (TAKARA), the qRT‐PCR assay was performed using SYBR Premix Ex Taq II (TAKARA) on an ABI QuantStudio 3 PCR System (Applied Biosystems, USA). Relative gene quantitation was determined by the 2‐^ΔΔCT^ method. The primer sequences for COL1*α*1, ALP, OPG, Runx2, OCN, Drp 1, SOD1, and GAPDH are listed in Table S1 (Supporting Information).

### Preparation of Extracts for Cell Culture

Samples extracts were collected according to the ISO 10993 standard. The sample discs were soaked in high glucose DMEM at a specific surface area ratio of 1.25 mL cm^−2^ and stood in a humidified incubator (37 °C, 5% CO_2_) for 24 h. Collect sample extracts and filter them with sterile filters for subsequent cell experiments. MC3T3‐E1 cells were cultured on confocal dishes for 24 h and then incubated with different sample extracts containing 33 × 10^−3^ m glucose to mimic peak hyperglycemia.

### Mitochondrial Morphology Observation

Mitochondrial morphology was visualized via MitoTracker Red (Molecular Probes, USA) following the manufacturer's protocol. After 24 h culture, the sample extracts were removed, and cells were further incubated in new serum‐free DMEM containing 100 × 10^−9^ m MitoTracker Red at 37 °C for 30 min. After rinsed gently and fixed with 4% PFA, cells were viewed with CLSM. Quantitative assessments of mitochondrial morphology and network were performed using the toolset MiNA (Mitochondrial Network Analysis) and MitoAnalyzer plugins on ImageJ (NIH, Bethesda, MD, USA).

### Mitochondrial Dynamics and Functions

Intracellular ROS production was evaluated by labeled with 2′,7′‐dichlorodihydrofluorescein diacetate (DCFH‐DA) (10 × 10^−6^ m; Thermo Scientific) for 30 min. The cells were counterstained with 2.5 × 10^−6^ m MitoSOX Red (Molecular Probes, a highly selective fluorescent dye targeting superoxide production in mitochondria in living cells) and 150 × 10^−9^ m Mitotracker Green (Molecular Probes) for 30 min at 37 °C to detect the mitochondrial ROS. The MMP of the cells was determined by 150 × 10^−9^ m TMRM (Molecular Probes) and 150 nM Mitotracker Green (Molecular Probes). After staining the cells for 30 min at 37 °C, they were photographed by CLSM. The corresponding fluorescent intensity was quantified and measured by the ImageJ software. Flow cytometry was further used to assess mitochondrial functions. Cells treated with different samples were collected into Eppendorf (EP) tubes and dyed with 2.5 × 10^−6^ m MitoSOX Red (Molecular Probes) or 50 nM TMRM (Molecular Probes) for 30 min respectively, detected by an Attune NxT flow cytometer (Thermo Scientific). Quantitative analysis was performed with FlowJo 10.6 software (Tree Star, Ashland, OR, USA).

### Diabetic Rat Bone Defects Mold Establishment

All animal experiments were conducted with the approval of the Institutional Animal Care and Use Committee of the West China Hospital, Sichuan University. The animals received humane care in compliance with the Guide for the Care and Use of Laboratory Animals. Eight weeks old healthy male Sprague Dawley (SD) rats were purchased from Dashuo Experimental Animal Center (Chengdu) and maintained in a specific pathogen‐free (SPF) animal facility with a 12 h light/dark cycle daily and regular sterile food and water. After fed adaptively for 1 week, rats received an intraperitoneal injection of streptozotocin (60 mg kg^−1^, Sigma), which dissolved in citrate buffer (pH 4.5). The tail vein blood was collected to record nonfasting blood glucose levels (BGL) weekly, employing an electronic glucometer (Accu‐Check Performa, Roche Diagnostics, USA). The criteria for success of the diabetic molding was defined as BGL > 16.7 mmol L^−1^. Those rats were excluded from this study because of experiencing failed molding or death.

### Surgical Procedure

Forty diabetic rats were randomly assigned to five groups: pristine PEEK group, the SPEEK group, the Zn‐SPEEK group, the Sr‐SPEEK group and the Zn/Sr‐SPEEK group. Rats were anesthetized by intraperitoneal injection of sodium pentobarbital to assure analgesia in the surgical site. After anesthesia, the hind limbs were shaved, and surgical procedures were carried out aseptically. Use a scalpel to make a 10 mm incision on the inside of the knee joint, and then the muscles were dissected to expose the femoral condyles and the tibias below the knee joint. The implant cavity (Φ2.3 mm) was created by a pilot drill of a dental handpiece with the irrigation of precooled sterilized physiological saline. Then, PEEK implants were implanted into the drilled holes gently, and soft tissues were sutured. Finally, the intramuscular injection of penicillin (100 000 IU) was used to prevent infection after surgery in the following 3 d. 4 and 8 weeks after surgery, the rats were euthanized by intraperitoneal injection of pentobarbital in overdose. The tibia specimens containing PEEK implants were harvested and fixed in 4% PFA solution before testing.

### Biomechanical Test

Biomechanical push‐out testing was performed to quantify osseointegration for various PEEK implants. All push‐out tests were tested by a material testing machine (Instron 8874, Norwood, USA). An axial compression load was applied to the implant at a loading rate of 5.0 mm min^−1^ and was stopped until the bone‐implant interface was destroyed. The failure load was regarded as the maximum push‐out load, and the load‐displacement curves were recorded. After the same processing procedure as *S. aureus* in *Antibacterial Assessment*, the implants separated from the pushed‐out tibia bone were subjected to FE‐SEM and EDS analyses.

### micro‐CT Analysis

The metaphysis of the femurs with implants was scanned by µ‐CT (SCANCO VivaCT80, Switzerland) at an isotropic voxel size of 15 µm, using the same energy setting of 70 kVp and an integration time of 300 ms. Raw images were reconstructed into serial cross‐sections and femoral histomorphometric parameters analyzed by SCANCO Medical Evaluation and Visualizer software. A hollow cylinder toward the bone marrow was defined as the volume of interest (VOI), including the 200 µm ring area extending from the implant periphery. BV/TV, Tb. N, Tb. Th, and Tb. Sp were calculated within the VOI. Besides, the BIC rate was also assessed.

### Histomorphometry and Immunohistochemistry (IHC)

After µ‐CT analysis, the bone specimens were sequentially dehydrated in a graded ethanol series (70–100%) and then embedded in a methyl methacrylate solution at 37 °C for 1 week to polymerize. Then, thin sections (≈5 µm in thickness) were prepared along the vertical to the implant using a microtome (Leica Microtome, Germany). Then, toluidine blue‐fuchsine stainings were used to detect the tissue response and bone ingrowth to the implanted materials. Osteoclasts were also analyzed through enzyme histochemistry in decalcified sections, employing tartrate‐resistant acid phosphatase (TRAP) staining.

Sections were further processed for standard immunofluorescent histochemical staining and analysis by incubating with primary antibodies (Abcam, MA, USA) against OPN, OCN, OPG, and DRP1 at 4 °C overnight. With a DAB‐horseradish peroxidase substrate system, detection of primary antibodies was visualized after incubation with secondary anti‐mouse IgG antibodies (Abcam). A fully electric automatic microscope (Nikon Eclipse Ni‐E, Japan) was used to capture imaging of the slice.

### Statistical Analysis

All data were presented as mean ± SD. Statistical analysis was performed using GraphPad Prism 8.0 software (GraphPad Software, Inc., CA, USA). Differences among groups were analyzed with One‐way analysis of variance (ANOVA) or Two‐way ANOVA, followed by Tukey's post hoc test for multiple comparisons, as detailed in respective figure legends. A *p*‐value of < 0.05 was considered to be statistically significant.

## Conflict of Interest

The authors declare no conflict of interest.

## Supporting information

Supporting Information

Supporting Video 1

## Data Availability

Research data are not shared.
