# Supplementary material for: Nutrient Element Decorated Polyetheretherketone Implants Steer Mitochondrial Dynamics for Boosted Diabetic Osseointegration
Source: Adv Sci (Weinh). 2021 Aug 16;8(20):2101778. doi: 10.1002/advs.202101778 (PMC8529468; doi:10.1002/advs.202101778)
Supplement: Supplementary file 1 — Supporting Information [file ADVS-8-2101778-s002.pdf]

WILEY-VCH

© 2020 Wiley-VCH GmbH

ADVANCED SCIENCE
Open Access

Supporting Information

for *Adv. Sci.,* DOI: 10.1002/advs.202101778

Nutrient Elements Decorated Polyetheretherketone Implants Steer Mitochondrial Dynamics for Boosted Diabetic Osseointegration

Hao Wang, Xinliang Fu, Jiacheng Shi, Limei Li, Jiyu Sun, Xidan Zhang, Qiuyang Han, Yi Deng, Xueqi Gan**

Supporting Information

Nutrient Elements Decorated Polyetheretherketone Implants Steer Mitochondrial Dynamics for Boosted Diabetic Osseointegration

Hao Wang, Xinliang Fu, Jiacheng Shi, Limei Li, Jiyu Sun, Xidan Zhang, Qiuyang Han, Yi Deng, Xueqi Gan**

H. Wang, X. Fu, J. Sun, X. Zhang, Dr. X. Gan
State Key Laboratory of Oral Diseases, National Clinical Research Center for Oral Diseases, West China Hospital of Stomatology, Sichuan University, Chengdu, 610041, China
Email: xueqigan@scu.edu.cn
J. Shi, Q. Han, Dr. Y. Deng
School of Chemical Engineering, State Key Laboratory of Polymer Materials Engineering, Sichuan University, Chengdu 610065, China
Email: dengyibandeng@scu.edu.cn
Dr. Y. Deng
Department of Mechanical Engineering, The University of Hong Kong, Hong Kong SAR, China
Dr. L. Li
Science and Technology Achievement Incubation Center, Kunming Medical University, Kunming, 650500, China

Captions of Figures

Figure S1. (A) SEM micrographs of ZnO and $Sr(OH)_2$ nanoparticles. (B) Distribution of the diameter of micro-pores on SPEEK surface. Distribution of the diameter of ZnO and $Sr(OH)_2$ nanoparticles on (C) Zn-SPEEK and (D) Sr-SPEEK, respectively ; (E) EDS spectra of the Zn&Sr-SPEEK; O 1s high-resolution spectra of (F) SPEEK and (G) Zn&Sr-SPEEK; (H) Zn and (I) Sr ion concentrations released from Zn-SPEEK, Sr-SPEEK, and Zn&Sr-SPEEK over 7 days, n = 3.

Figure S2. Morphologies of cells cultured on various samples for 7 day captured by SEM (Red asterisk, poorly spread cells; yellow asterisk, fully spread cells).

Figure S3. Representative distribution of (A) mROS stained with MitoSox and (B) MMP stained with TMRM analyzed by flow cytometry. Percentage of (C) MitoSox-positive and (D) TMRM-positive cells, respectively, n = 3. (One-way ANOVA and Tukey's post hoc test; *, #, +, and ++ represent $p < 0.05$ when compared with PEEK, SPEEK, Zn-SPEEK, Sr-SPEEK, respectively.)

Figure S4. (A) Micro-CT reconstruction and (B) X-ray images of the sagittal plane at 4 weeks.

Figure S5. The bone attachment and bone ingrowth onto the implant surface at 4 weeks observed by SEM (Red arrow point to the scratches resulted from sandpaper grinding, green arrows reveal the calcified tissues and blue arrows indicate the collagen).

Figure S6. The 3D reconstructed micro-CT images of newborn bone (A) with implants and (B) without implants at 4 weeks (Implant, yellow; new bone, purple).Representative images of (C) Tb.Th and (D) Tb.Sp at 4 weeks.

Video S1. Rotation of 3D reconstructions from Z-axis stacks of confocal images after 3 days of cell culture.

Table S1. Primer sequences for real-time qPCR analysis of the mRNA expression.

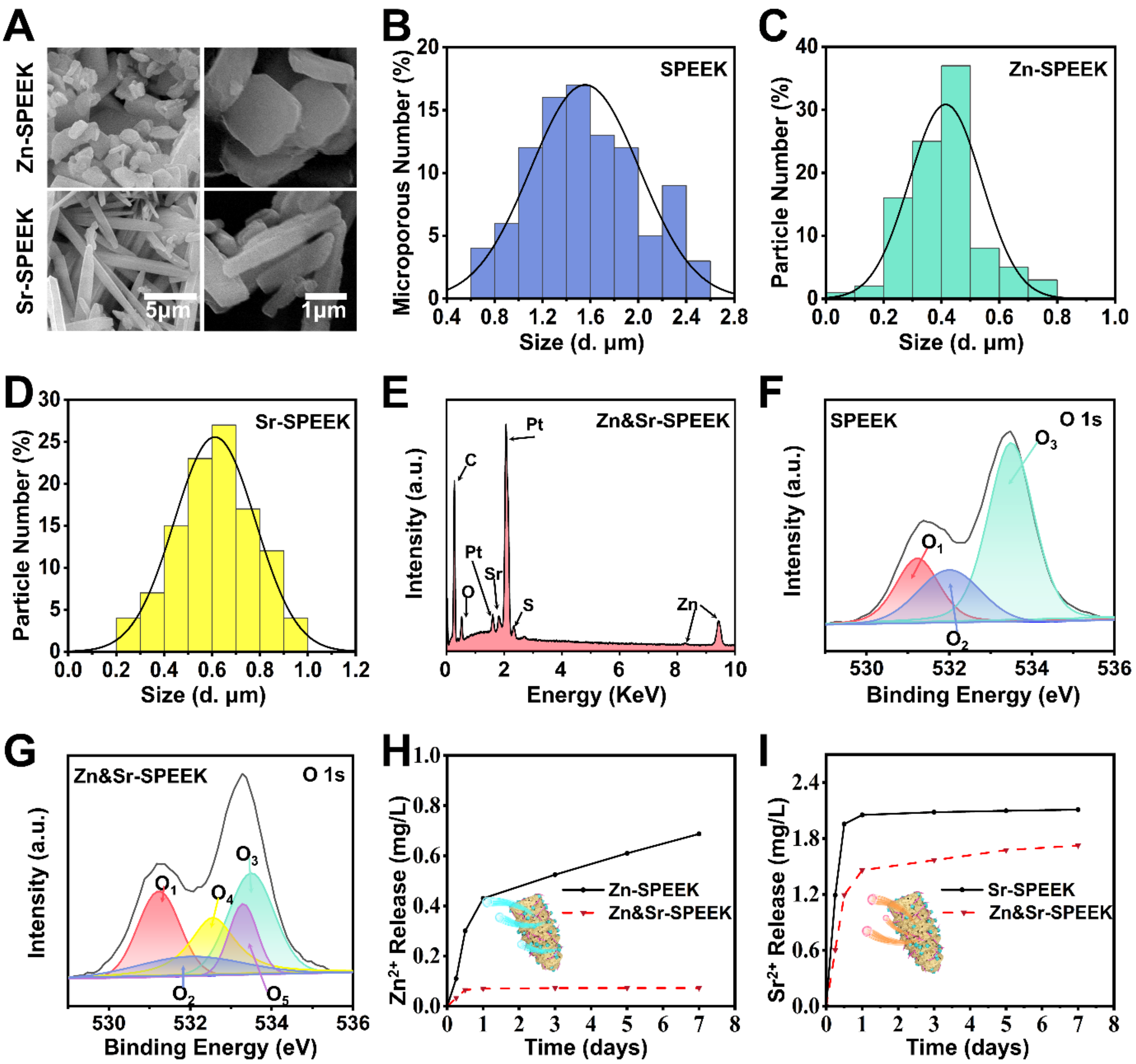

Figure S1. (A) SEM micrographs of ZnO and $Sr(OH)_2$ nanoparticles. (B) Distribution of the diameter of micro-pores on SPEEK surface. Distribution of the diameter of ZnO and $Sr(OH)_2$ nanoparticles on (C) Zn-SPEEK and (D) Sr-SPEEK, respectively ; (E) EDS spectra of the Zn&Sr-SPEEK; O 1s high-resolution spectra of (F) SPEEK and (G) Zn&Sr-SPEEK; (H) Zn and (I) Sr ion concentrations released from Zn-SPEEK, Sr-SPEEK, and Zn&Sr-SPEEK over 7 days, n = 3.

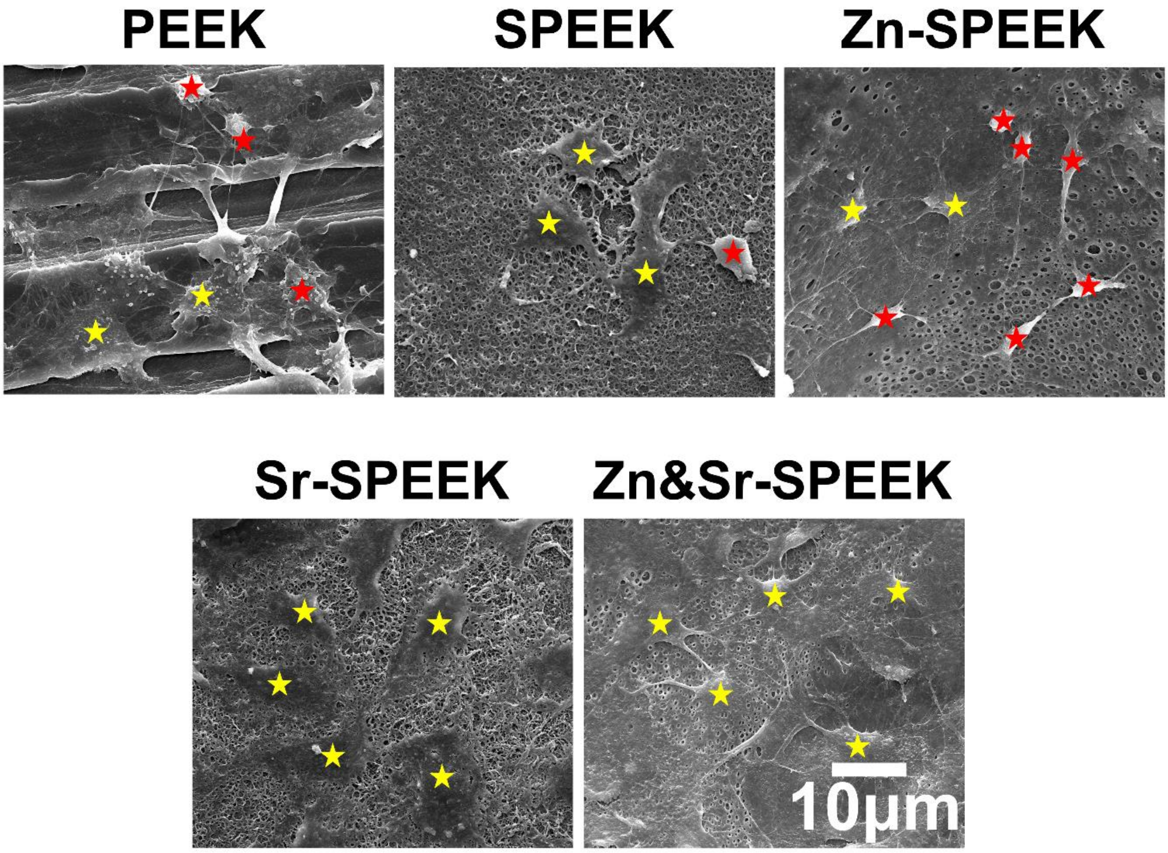

Figure S2. Morphologies of cells cultured on various samples for 7 day captured by SEM (Red asterisk, poorly spread cells; yellow asterisk, fully spread cells).

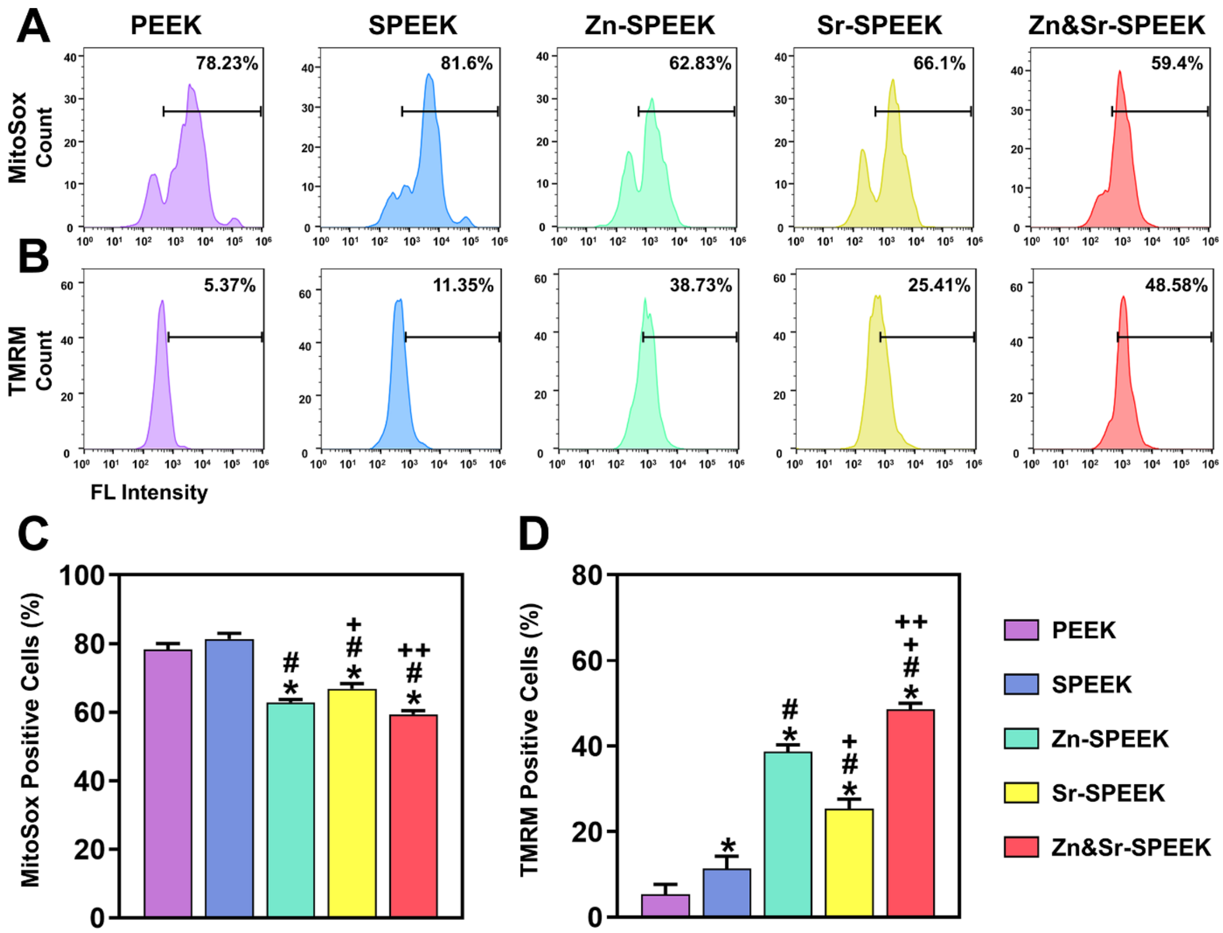

Figure S3. Representative distribution of (A) mROS stained with MitoSox and (B) MMP stained with TMRM analyzed by flow cytometry. Percentage of (C) MitoSox-positive and (D) TMRM-positive cells, respectively, n = 3. (One-way ANOVA and Tukey's post hoc test; *, #, +, and ++ represent $p < 0.05$ when compared with PEEK, SPEEK, Zn-SPEEK, Sr-SPEEK, respectively.)

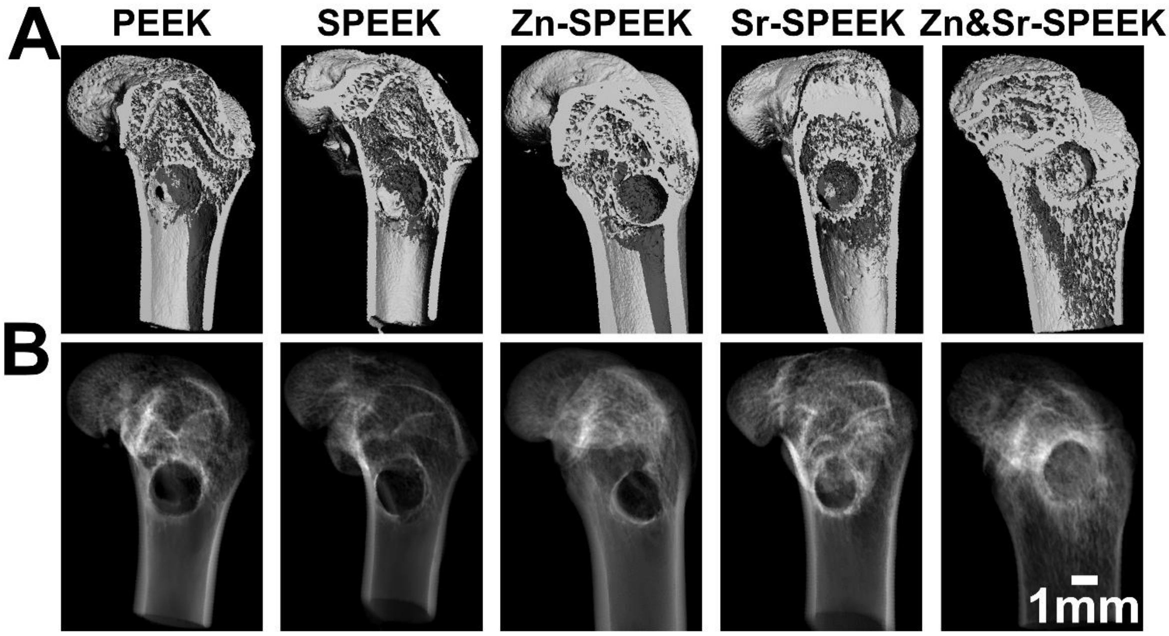

Figure S4. (A) Micro-CT reconstruction and (B) X-ray images of the sagittal plane at 4 weeks postoperatively.

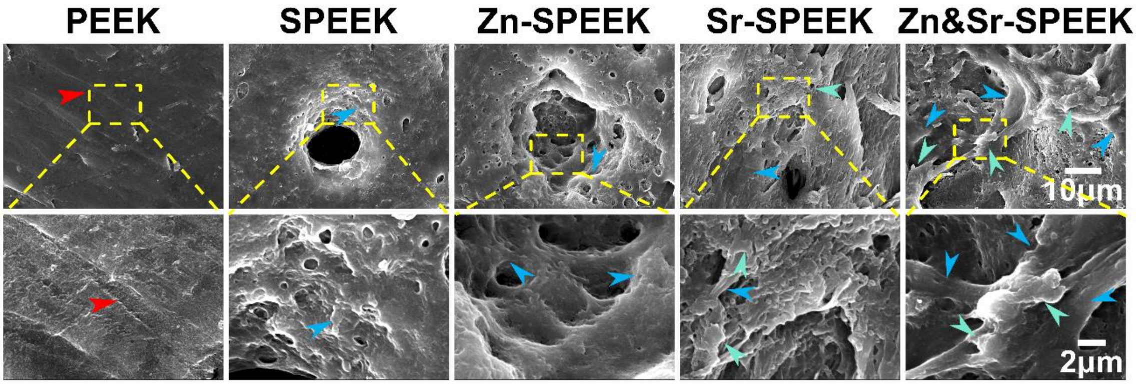

Figure S5. The bone attachment and bone ingrowth onto the implant surfaces at 4 weeks observed by SEM (Red arrow point to the scratches on pristine PEEK implant, green arrows reveal the calcified tissues and blue arrows indicate the collagen deposited on the implants).

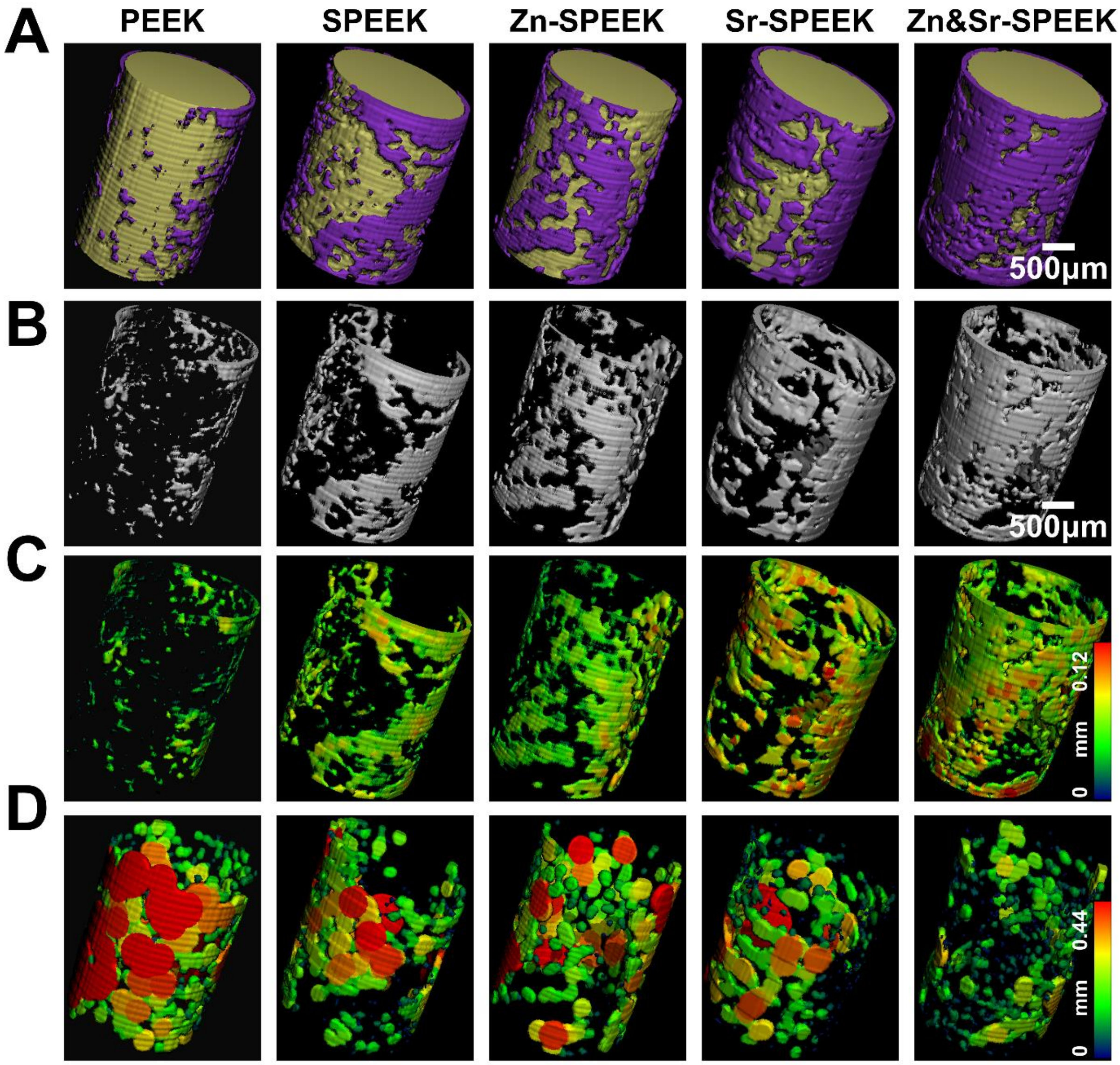

Figure S6. The 3D reconstructed micro-CT images of newborn bone (A) with implants and (B) without implants at 4 weeks (Implant, yellow; new bone, purple). Representative images of (C) Tb.Th and (D) Tb.Sp at 4 weeks.

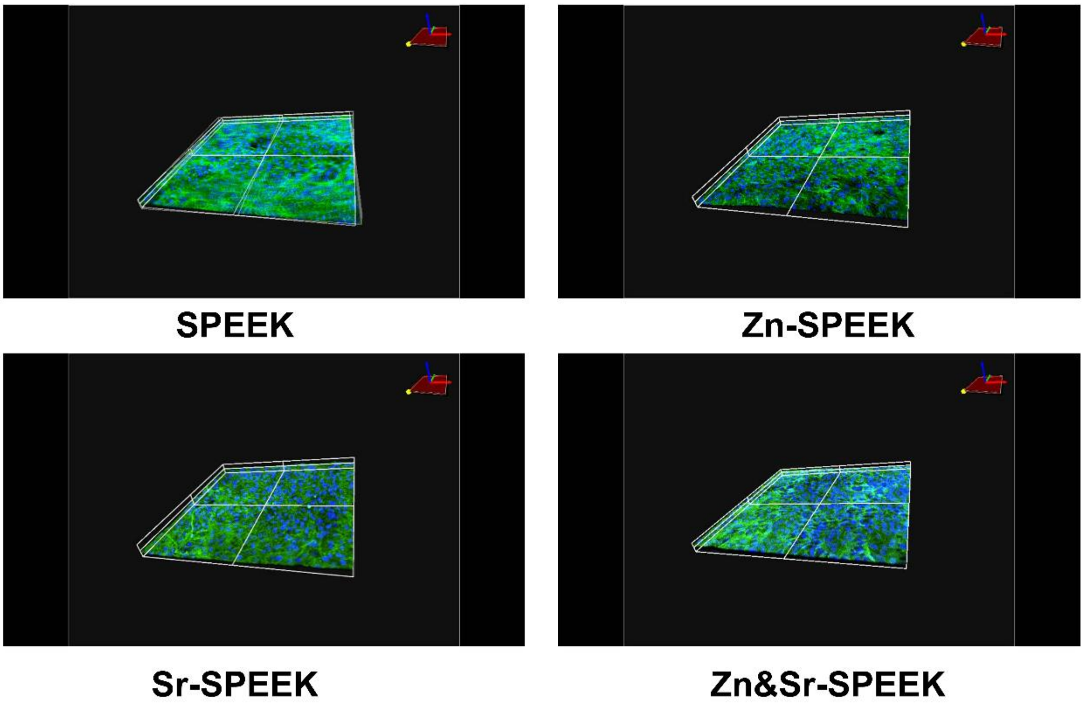

Video S1. Rotation of 3D reconstructions from Z-axis stacks of confocal images after 3 days of cell culture.

Table S1. Primer sequences for real-time qPCR analysis of the mRNA expression.

Gene	Primers (5′-3′)	
RUNX2	F: CCGGGAATGATGAGAACTA	R: GGACCGTCCACTGTCACTTT
ALP	F: GCTGATCATTCCCACGTTTT	R: CTGGGCCTGGTAGTTGTTGT
COL1α1	F: AGAGCATGACCGATGGATT	R: TGAGCTCGATCTCGTTGGATR
OCN	F: GAACAGACTCCGGCGCTA	R: AGGGAGGATCAAGTCCCG
OPG	F: GTTTCCCGAGGACCACAAT	R: CCATTCAATGATGTCCAGGAG
DRP1	F: CCGGGAATGACCAAAGTACC	R: TGGGATTACTGATGAACCGAAGA
SOD1	F: CACTCTAAGAAACATGGTGG	R: GATCACACGATCTTCAATGG
GAPDH	F: ACTTTGTCAAGCTCATTTCC	R: TGCAGCGAACTTTATTGATG

F: forward; R: reverse.
