# Supplementary figures and images for: Nutrient Element Decorated Polyetheretherketone Implants Steer Mitochondrial Dynamics for Boosted Diabetic Osseointegration
Source: Adv Sci (Weinh). 2021 Aug 16;8(20):2101778. doi: 10.1002/advs.202101778 (PMC8529468; doi:10.1002/advs.202101778)

## Slide 1
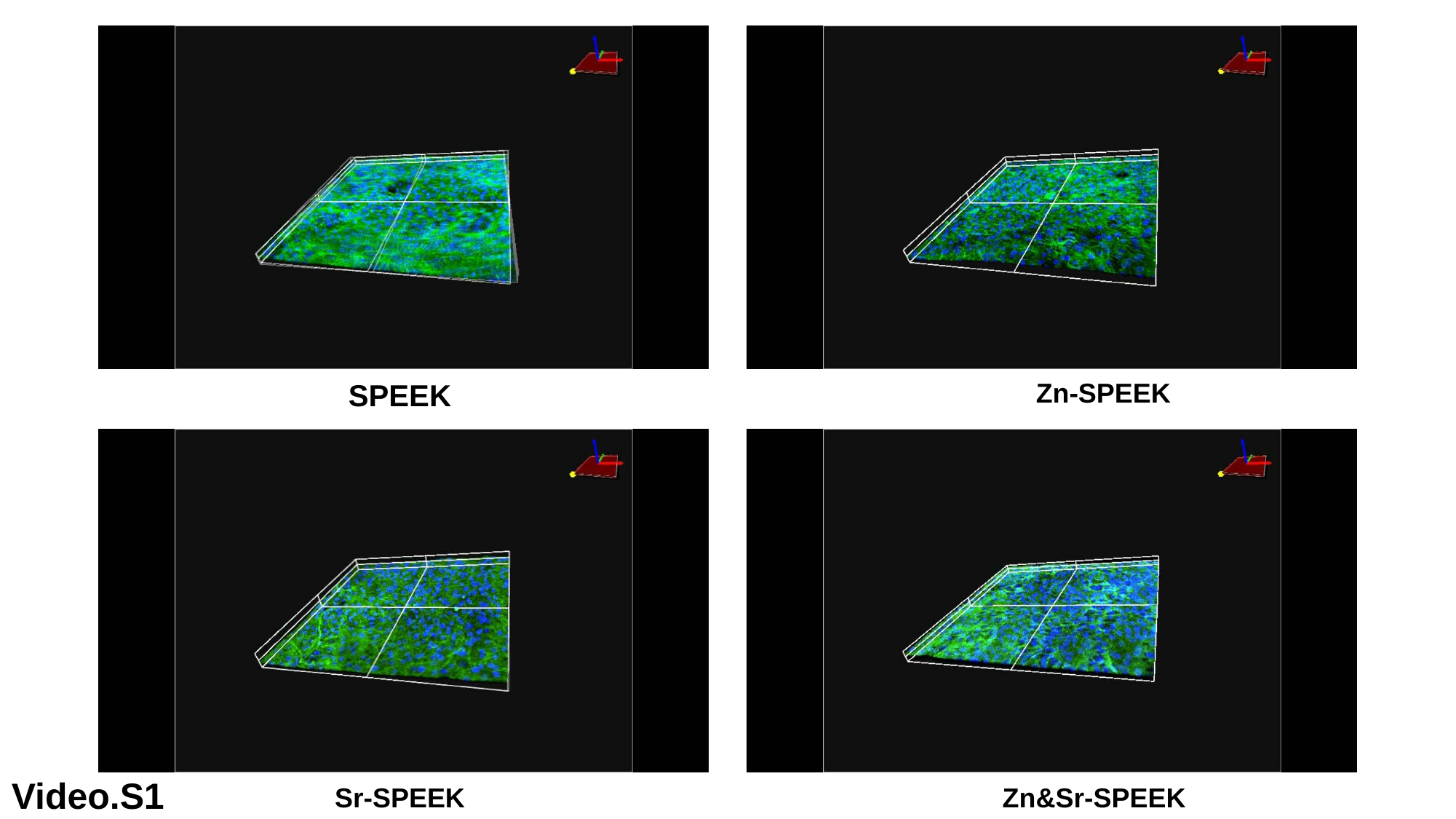

SPEEK
Zn-SPEEK
Video.S1
Sr-SPEEK
Zn&Sr-SPEEK

Supplement: Supplementary file 2 — Supporting Video 1 [file ADVS-8-2101778-s001.pptx]
